# A New Bis‐Urea Based Cage Receptor for Anions: Synthesis, Solid State Structures and Binding Studies

**DOI:** 10.1002/asia.202401258

**Published:** 2024-12-06

**Authors:** Daniele Paderni, Mauro Formica, Eleonora Macedi, Luca Giorgi, Patrizia Rossi, Michele Retini, Nicola De Cata, Giovanni Zappia, Giovanni Piersanti, Vieri Fusi

**Affiliations:** ^1^ Department of Pure and Applied Sciences University of Urbino Carlo Bo via Ca' le Suore 2–4 61029 Urbino Italy; ^2^ Department of Industrial Engineering University of Firenze via Santa Marta 3 50139 Firenze Italy; ^3^ Department of Biomolecular Sciences University of Urbino Carlo Bo via Ca' le Suore 2–4 61029 Urbino Italy; ^4^ Dipartimento di Promozione delle Scienze Umane e della Qualità della Vita University San Raffaele via di Val Cannuta 247 Rome Italy

**Keywords:** Anions coordination, Aza-macrocycle, Cage compounds, Norfloxacin Urea

## Abstract

The synthesis and characterization of a novel bis‐urea‐based cage receptor for anions (3*S*,15*S*)‐3,15,20,25‐tetramethyl‐1,4,6,12,14,17,20,25‐octaazatricyclo[15.5.5.17.11]octacosa‐7(28),10‐diene‐2,5,13,16‐tetraone (**L**) is reported. **L** is a macro‐bicyclic ligand built on the 1,7‐dimethyl‐1,4,7,10‐tetraazacyclododecane scaffold to obtain a cage topology in which two ureido moieties have been inserted as binding sites for anions. **L** can interact with anion guests (G) via H‐bonding; in particular, it binds both spherical (Cl^−^) and V‐shaped anions (AcO^−^) as well as more complex carboxylate anions, such as the norfloxacin (Nor^−^). NMR experiments highlight that the interaction between **L** and G mainly occurs at the ureido moieties. **L** forms **L**‐G adducts of 1 : 1 ([**L**G]^−^) and 1 : 2 ([**L**G_2_]^2−^) stoichiometry with Cl^−^ and AcO^−^. Otherwise, in the case of Nor^−^ only the formation of the [**L**G]^−^ complex is observed. **L** shows higher formation constants values for [**L**AcO]^−^ (2.9) and [**L**Nor]^−^ (3.6) than [**L**Cl]^−^ suggesting a stronger interaction with the carboxylate anions. In the solid state, three crystal structures of the H**L**⋅G species were obtained (G=Cl^−^, AcO^−^, ClO_4_^−^) highlighting the H‐bonding interaction between the chloride, acetate or perchlorate anions and the −NH functions of the ureido fragment. The comparison between the two parent open chain receptors (**Lb‐c**) and **L** has been reported and discussed.

## Graphical abstract

A New Bis‐Urea Based Cage Receptor for Anions was synthesized. NMR experiments showed the ability of the new system to interact with anions via H‐bonding; in particular, it binds both spherical (chloride) and V‐shaped anions (acetate and norfloxacin). Results highlight that the interaction between the ligand and anions mainly occurs at the ureido moieties, showing a stronger interaction with the carboxylate anions.

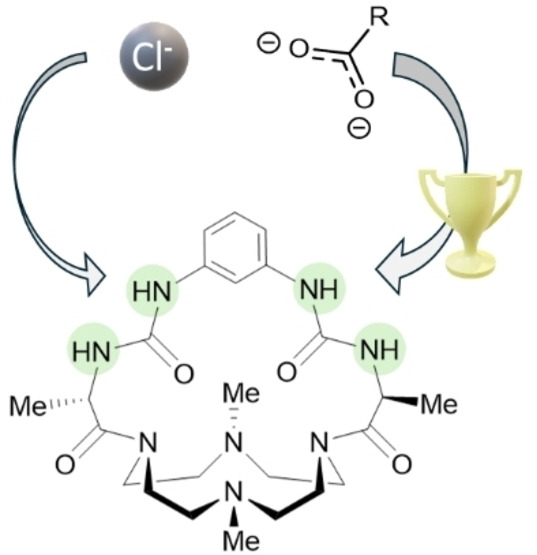

## Introduction

In the recent decades, synthetic ligands for anions have attracted great interest in the field of supramolecular chemistry.[^1^, ^2^, ^3^, ^4^, ^5^, ^6^, ^7^] The reason for this lies in both the relevance of anions in biological processes and environment,^[8]^ and the potential applications of these systems as chemosensors,^[9]^ organic catalysts^[10]^ and membrane carriers.^[11]^

Several strategies can be exploited in the design of receptors for anions, mainly differing in the nature of the host‐guest interaction, namely: i) *via* classical coordination chemistry, by employing a metal complex with an unsaturated metal core;[^12^, ^13^, ^14^] ii) *via* charge‐charge interaction, by using species with a permanent positive charge;[^15^, ^16^, ^17^] iii) *via* hydrogen bond formation, by means of neutral molecules with hydrogen donor groups.[^18^, ^19^] In the latter case, urea‐based ligands have been largely exploited due to the acidity of the −NH functions caused by the electron‐withdrawing −C=O groups, which provide the ability to form strong hydrogen‐bonding interactions with negatively charged species.^[20]^ In particular, the insertion of urea groups provides the receptors with directional binding sites for anions under neutral conditions, behaving as double H‐bond donors through a complementary interaction with the oxygen atoms of the oxyanions (*e. g*. carboxylates, phosphates etc.), and as bifurcated H‐bond donors with the spherical halide anions.^[21]^

**Figure 1:**
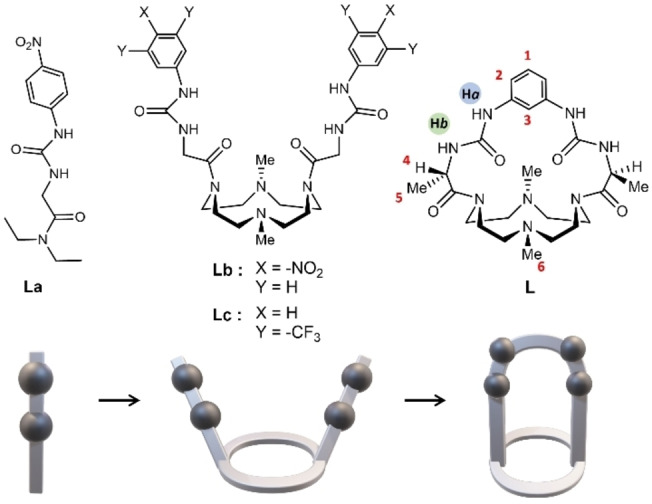
Structures of ligand **L** and its parent compounds **La‐c**, together with ^1^H NMR labelling scheme for **L** (top) and schematic representation of the structures (bottom).

Among all factors that modulate the binding affinity in the “Host‐Guest” association complexes, such as the ionic strength, nature of the anion, counterion, solvent and type and strength of the interaction involved, the complementarity and preorganization have become central and crucial challenges in the design of synthetic receptors for anions. The reason for this lies in ensuring selective systems in which the binding sites of the host are structurally complementary to the guest.[^22^, ^23^, ^24^] In line with these objectives, many groups working in this field have focused their efforts on the design and synthesis of both macrocycles and cages.[^25^, ^26^, ^27^, ^28^, ^29^, ^30^, ^31^, ^32^, ^33^, ^34^, ^35^, ^36^, ^37^, ^38^, ^39^, ^40^, ^41^, ^42^, ^43^] These fully organic molecular systems possess pockets with unique shape and size and can be provided with specific functional groups pointing inward the cavity, offering advantages in terms of selectivity towards specific guests and stability of the complexes. As a result, these improvements have opened the horizon to several applications, such as molecular recognition and encapsulation, catalysis, gas separation and gas sorption.^[44]^ Recently, we reported the synthesis of three urea based hosting molecules for anions, (**La**), (**Lb**) and (**Lc**),[^45^, ^46^] whose structures are reported in Figure 1. **La** is a linear molecule with a *p*‐nitrophenylureido group, while **Lb** and **Lc** are the result of a design focused to increase the molecular complexity of the parent ligand **La**. Indeed, the latter are constituted by a macrocyclic 1,7‐dimethyl‐1,4,7,10‐tetraazacyclododecane (Me_2_[12]aneN_4_) polyamine base functionalized in *trans*‐positions with two *p*‐nitrophenylureido (**Lb**) or two 3,5‐bis(trifluoromethyl)phenylureido (TFP‐ureido) groups (**Lc**). ^1^H NMR and UV‐Vis spectroscopy studies performed in DMSO – 0.5 % water solution showed the ability of the three receptors to bind guests (G) such as AcO^−^ and Cl^−^ *via* the formation of H‐bonds with the urea functions, whereas they were not able to bind I^−^ and Br^−^. The replacement of the nitrophenyl group with TFP functions in **Lc** brought about several advantages including: i) the ability to interact with F^−^ avoiding the deprotonation of the urea groups, also in the presence of high concentration of the anion added; ii) the enhancement of the solubility in common organic solvents such as CH_3_CN, where **Lc** showed the ability to interact also with Br^−^; iii) the improvement of the optical properties given by the TFP‐ureido chromophore, which allows for an optical response in the presence of AcO^−^ by a notable change also in the visible range and finally d) the ability of this fragment to increase NH polarization of the urea and prevent aggregation/self‐association phenomena.[^35^, ^36^] By comparing the results of ^1^H NMR titrations performed in DMSO‐*d_6_* ‐ 0.5 % D_2_O solution on all ligands with AcO^−^, differences could be observed in the stoichiometry and binding constant values. **La** forms only the 1 : 1 [LG]^−^ species, while **Lb** and **Lc** form both the 1 : 1 [LG]^−^ and 1 : 2 [LG_2_]^2−^ species. Interestingly, the formation constant values for the [LAcO]^−^ species were found to be higher for **Lb** and **Lc** than for **La** ([**Lb**AcO]^−^ log *K*=5.6; [**Lc**AcO]^−^ log *K*=4.5; [**La**AcO]^−^ log *K*=2.9),[^45^, ^46^] suggesting that in **Lb** and **Lc** the urea groups of the side‐arms cooperate in the stabilization of the guest. All results combined, these observations evidenced the crucial role of the tetraaza‐macrocycle in the preorganization of the host, allowing for the cooperation of the two urea‐based side arms in the anion binding.

Following these results, a new class of receptors has been designed with the aim to increase the preorganization of the receptor by linking the two ureido binding sites present in **Lb**‐**c**, affording a macro‐bicyclic cage. Within this scope, herein the synthesis and characterization of a macro‐bicyclic ligand based on the Me_2_[12]aneN_4_ scaffold **L** ((3*S*,15*S*)‐3,15,20,25‐tetramethyl‐1,4,6,12,14,17,20,25octaazatricyclo[15.5.5.17.11] octacosa‐7(28),10‐diene‐2,5,13,16‐tetraone) is reported, in which the two ureido side‐arms have been linked and spaced by a phenyl ring (Figure 1). To the best of our knowledge, **L** is the first reported tetraazacyclododecane‐based cage containing two urea groups in the macrocyclic framework. In this case the glycine moiety, exploited as linker in the synthesis of the non‐chiral and/or symmetrical **La‐c** ligands, has been replaced by the chiral amino acid L‐alanine, thus introducing chirality into the cage molecule **L** and adding new possible functionalities.

The ability of the C2‐symmetrical **L** to interact with anions was studied both in solution and in the solid state through, respectively, ^1^H NMR measurements and single crystal X‐ray diffraction analysis of three structures containing **L** and different anions. All the studies were aimed at understanding the effect of the stiffening of the structure, obtained both by macrocyclization of the two ureido side arms as well as using a more steric hindered amino‐acid as linker, on the binding properties of the receptor towards anionic species.

## Results and Discussion

### Synthesis

**Scheme 1:**
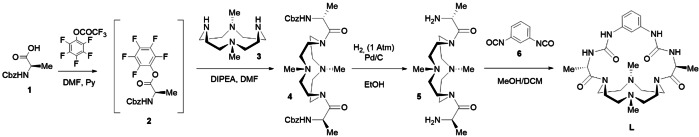
Synthesis of the ligand **L**.

The new C_2_‐symmetrical bis‐urea cage receptor **L** was prepared by the reaction of 1 equiv. of free primary diamine **5** with 1 equiv. of commercially available 1,3‐Diisocyanatobenzene (**6**) (Scheme 1) in high‐dilution technique.^[47]^ The crude product was readily purified by flash chromatography on silica gel. As expected, as in most cases, the cyclization reaction resulting in macrocycles proceeds poorly at an isolated yield of 46 % on account of the closure of the ring competes with the formation of linear oligomers. To overcome this issue, template techniques were also utilized^[48]^ with the addition of 1 equiv. of chloride and sodium ions, but in our case the yield of this macrocyclic product did not increase. This led us to the assumption that **5** is already in a folded conformation i. e. U‐turn bent orientation (probably due to orthogonal dipolar interactions between amide C=O bond dipoles) before the macrocyclization initiated, which is much more favourable for the cyclization reaction in comparison with the linear polymerization. The key intermediate diamine **5** was achieved by simple hydrogenolysis (1 atm Hydrogen on Pd/C catalyst) of Cbz‐carbamate protecting groups precursor **4**, which was obtained by amide coupling of 1,7‐dimethyl‐1,4,7,10‐tetraazacyclododecane **3** with pentafluorophenyl activated ester **2** of commercially available N‐Cbz‐alanine **1**.

amide coupling of 1,7‐dimethyl‐1,4,7,10‐tetraazacyclododecane **3** with pentafluorophenyl activated ester **2** of commercially available N‐Cbz‐alanine **1**. We chose the pentafluorophenyl activated ester of **1**, as we have had previous success forming achiral anion receptors[^45^, ^46^] and chiral anion‐binding catalysis^[49]^ with it and its ease of preparation (Scheme 1). The choice to introduce the small, monovalent, and lipophilic methyl group i. e. the use of L‐alanine as a chiral pool, was motivated by the fact that this group has the appropriate characteristics including the displacement of water molecules during molecular recognition, i. e., the realization of hydrophobic and van der Waals interactions; the modulation of physicochemical properties; and the control of the conformational properties of a given scaffold to both preserve the binding properties and favour the buildup of a well‐organized structure. Scheme 1 outlines the synthetic approach for the easy and scalable preparation of cage bis‐urea receptor **L**.

### Solid State Structures

**Figure 2:**
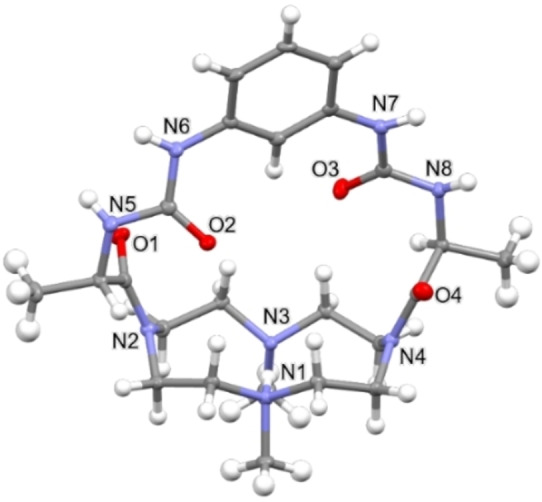
Ortep‐3 view of the H**L** ^+^ cation in of H**L**⋅AcO⋅2.5H_2_O (**7**). Ellipsoid probability=30 %.

Three isomorphous crystal structures of **L** containing acetate (H**L**⋅AcO⋅2.5H_2_O, **7**), perchlorate (H**L**⋅ClO_4_⋅H_2_O⋅MeOH, **8**) and chloride (H**L**⋅Cl⋅2.5H_2_O, **9**) anions were obtained by slow evaporation of methanol solutions containing a mixture of **L** and anion guests. Single crystal X‐ray diffraction of the three compounds revealed that in all asymmetric units a monoprotonated H**L** ^+^ cation is present. In addition, compound **7** exhibits one acetate anion and 2.5 water molecules, compound **8** one perchlorate anion, one water molecule and one methanol molecule and compound **9** one chloride anion and 2.5 water molecules. Crystallographic data and refinement parameters are reported in Table 1 while in Figures 2 and S1 ORTEP views of H**L** ^+^ cation in **7**, **8** and **9** are reported.

**Table 1:** Crystallographic data and refinement parameters for **7**, **8** and **9**.

	**7**	**8**	**9**
empirical formula	C_26_H_47_N_8_O_8.5_	C_25_H_45_ClN_8_O_10_	C_24_H_44_ClN_8_O_6.5_
formula weight	607.71	653.14	584.12
T (K)	150	110	150
Crystal system, space group	Monoclinic, P2_1_	Monoclinic, P2_1_	Monoclinic, P2_1_
λ (Å)	0.71073	1.54178	0.71073
Unit cell dimensions (Å, °)	a=8.9970(4), b=14.5298(7), c=12.0176(6), β=103.199(5)	a=8.7126(3), b=4.6388(5), c=12.0508(4), β=98.234(2)	a=8.841(1), b=14.161(1), c=11.999(1), β=101.62(1)
*V* (Å^3^)	1529.5(1)	1521.14(9)	1471.6(3)
Z, d_calc_(g/cm^3^)	2, 1.320	2, 1.426	2, 1.318
μ (mm^−1^)	0.100	1.701	1.318
F(000)	654	696	626
Reflections collected/unique	12339/6699	36481/5578	17149/8902
Data/ parameters	6699/421	5578/547	8902/394
Final R indices [I>2σ(I)]	R1=0.0694, wR2=0.0804	R1=0.0388, wR2=0.0975	R1=0.0728, wR2=0.1615
R indices all data	R1=0.1234, wR2=0.0949	R1=0.0406, wR2=0.0989	R1=0.1135, wR2=0.1981
GoF	0.977	1.058	1.030

**Figure 3:**
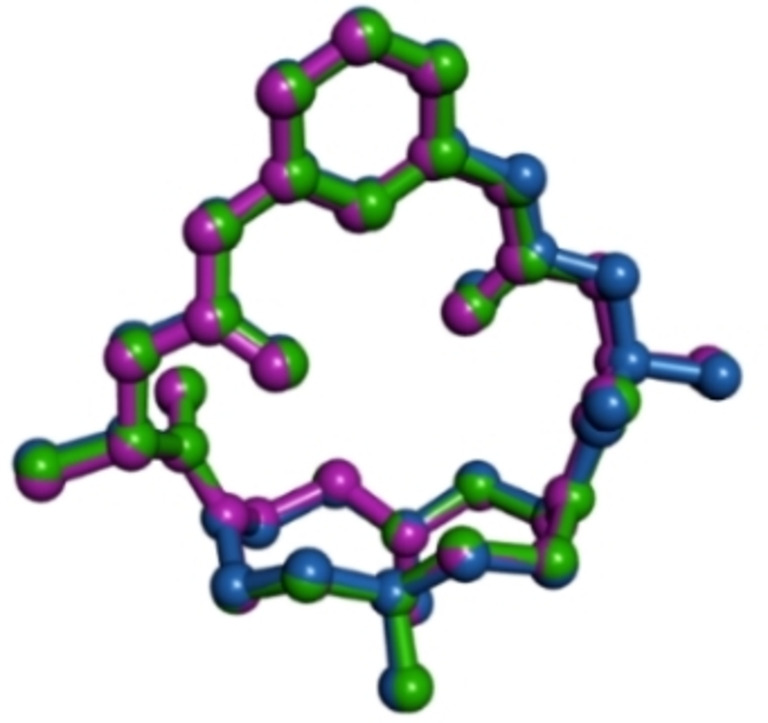
Superimposition of the H**L** ^+^ cation in **7** (green), **8** (pink) and **9** (pale blue). Hydrogen atoms have been omitted.

As evidenced in Figure 3, the conformation taken by the H**L** ^+^ cation in the three structures is well comparable.

The macrocyclic base has the common, for mono‐protonated cyclen derivatives, [2424]‐C corners conformation,^[50]^ with the two facing nitrogen atoms N1 and N3 involved in a strong hydrogen bond (Table 2).^[51]^ The aromatic ring of the bridging chain is almost perpendicular to the mean plane defined by the four nitrogen atoms of the macrocyclic base (the angle between this plane and the one defined by the carbon atoms of the aromatic ring is 78.0(1) in **7**, 76.6(1) in **8** and 75.8(1)° in **9**), while the distance between the hydrogen atom H20 (the hydrogen atom of the aromatic ring pointing toward the macrocyclic base, see Figure S2) and the mean plane defined by the four nitrogen atoms of the macrocyclic base is about 4.5 Å (Table S1).

**Table 2:** Selected H‐bond interactions in **7**, **8** and **9**.

D‐H⋅⋅⋅A	D⋅⋅⋅A (Å)	H⋅⋅⋅A (Å)	D−H⋅⋅⋅A (°)
**7**			
O1W−H1WB⋅⋅⋅O1M	2.736(6)	1.85(4)	173(3)
O3W−H3WA⋅⋅⋅O1M	2.72(1)	1.9(1)	167(1)
N1‐H1N⋅⋅⋅N3	2.753(5)	1.80(3)	176(3)
N6‐H6N⋅⋅⋅O2M	2.720(6)	1.88(5)	165(4)
N7‐H7N⋅⋅⋅O2W	2.903(6)	2.02(4)	167(4)
O3W−H3WB⋅⋅⋅O1^1^	2.809(9)	1.97(7)	169(7)
O1W−H1WA⋅⋅⋅O1^1^	2.756(6)	1.90(5)	174(4)
N8‐H8N⋅⋅⋅O1W^2^	3.114(6)	2.28(4)	155(4)
O2W−H2WA⋅⋅⋅O1W^2^	2.725(7)	1.88(6)	165(5)
O2W−H2WB⋅⋅⋅O1M^3^	3.227(6)	2.41(4)	163(3)

**8**			
N1‐H1N⋅⋅⋅N3	2.748(4)	1.62(4)	179(4)
O1W−H1WB⋅⋅⋅O1	2.767(4)	1.90(5)	166(5)
O1M−H1OM⋅⋅⋅O1W	2.784(5)	1.89(6)	155(5)
N5‐H5N⋅⋅⋅O14B/O14C^1,*^	3.169(9)/ 3.03(1)	2.36(5)/ 2.19(5)	157(4)/167(4)
O1W−H1WA⋅⋅⋅O13A/O13B^4,*^	2.78(2)/2.943(8)	2.01(6)/ 2.16(6)	163(6)/164(5)
N6‐H6N⋅⋅⋅O1M^4^	2.841(5)	2.10(5)	159(5)
N5‐H5N⋅⋅⋅O13A/O13B/O13C^4^	2.92(3)/ 3.169(9)/ 3.03(1)	2.07(6)/ 2.36(5)/ 2.19(5)	170(5)/ 157(4)/ 167(4)
N7‐H7N⋅⋅⋅O12A/O12B/O12C^5,*^	3.25(2)/ 3.01(1)/ 3.08(1)	2.36(5)/ 2.10(5)/ 2.21(5)	158(4)/ 165(4)/ 156(4)
N8‐H8N⋅⋅⋅O1W^3^	3.034(4)	2.29(5)	156(5)

**9**			
N1‐H1N⋅⋅⋅N3	2.715(6)	1.79(4)	172(4)
N7‐H7N⋅⋅⋅O1W	2.870(5)	1.98(3)	177(3)
O1W−H1WA⋅⋅⋅Cl1	3.377(5)	2.60(6)	148(4)
N5‐H5N⋅⋅⋅Cl1^3^	3.414(5)	2.60(5)	149(4)
N6‐H6N⋅⋅⋅Cl1^3^	3.256(5)	2.42(5)	157(4)
O1W−H1WB⋅⋅⋅O2W^6^	2.700(7)	1.87(7)	167(6)
N8‐H8N⋅⋅⋅O2W^6^	3.111(5)	2.30(4)	149(4)
O2W−H2WB⋅⋅⋅Cl1^7^	3.193(5)	2.33(4)	166(3)
O3W−H3WA⋅⋅⋅O1^8^	2.858(9)	2.00(8)	170(6)

In all the three structures the hydrogen atom H20 is involved in a weak hydrogen bond with one C=O group (the one containing O3) giving rise to an almost planar six membered ring (Figure S3, C20‐H20⋯O3: **7**=2.185(3) Å (H20⋯O3), 121.9(3)°; **8**=2.20(5) Å (H20⋯O3), 122(4)°; **9**=2.184(3) Å (H20⋯O3), 123.2(3)°) the maximum deviation from the plane is always due to C21: 0.208(5), 0.168(5), 0.102(5) Å in **7**, **8** and **9**, respectively.

Concerning the disposition of the four C=O groups, it can be observed that the closest to the macrocyclic base (O1 and O4) point outward the cage, while the other two (O2 and O3) point inward the cavity (Figure S4).

The overall shape of the H**L** ^+^ macrocycle cavity may be described considering the [2424]‐C corners “rectangular” shape adopted by the base and the reciprocal disposition (out‐in‐in‐out) of the four carbonyl oxygen atoms (see Figure S4). Finally, the dimensions of the cavity were defined by measuring the distances between the nitrogen atoms of the macrocycle base and the oxygen atoms of the chain (see Figure S2 and Table S1 for the retrieved values).

Concerning the intermolecular interactions, neither O2 nor O3 are involved in intermolecular H‐bonds (Figure S4 and Table 2), whereas O1 acts as an acceptor in strong hydrogen bonds and O4 appears involved in weak C‐H⋅⋅⋅O interactions. C11=O1 acts as a bifurcated acceptor interacting with two water molecules (in both **7** and **9**) (Figure S5).

Concerning the N−H groups of the chain in **7** and **9**, the upper ones (N6 and N7, see Figure 2) interact, *via* strong hydrogen bonds, with a water molecule (N7) and either a carboxyl oxygen atom (in **7**) or a Cl^−^ anion (in **9**) (N6, see Figure S5). The other two nitrogen atoms (N5 and N8) are involved instead in weaker H‐bond interactions. In fact, in **7N**5‐H5 weakly interacts with the acetate oxygen atom O2 M (H5⋅⋅⋅O2 M 2.53(1) Å, N5‐H5⋅⋅⋅O2 M 135(2)°) while in **9** this group interacts with the chloride atom Cl1 (H5⋅⋅⋅Cl1 2.60(1) Å, N5‐H5⋅⋅⋅Cl1 149(2)°). The last nitrogen atom of the chain, N8, weakly interacts in both cases with a water molecule (Table 2).

Notably, **8** shows a very different pattern compared to **7** and **9**. In fact, in this case: i) the oxygen atom O1 does not behave as a bifurcated acceptor, interacting with just one water molecule (Figure S6); ii) the nitrogen atom N6 interacts with the O−H group of a methanol molecule (Figure S6); iii) the nitrogen atom N8 gives rise to a strong hydrogen bond with an oxygen atom of a water molecule (Figure S6), while the N5 and N7 atoms interact with a perchlorate anion.

In fact, although the independent ClO_4_^−^ anion is disordered, all the three refined models (which have similar occupancy factors) interact with two oxygen atoms (O12 and O13) with the hydrogen atoms H7N and H5N (see Table 2 and Figure S1).

### Solution Studies

**Figure 4:**
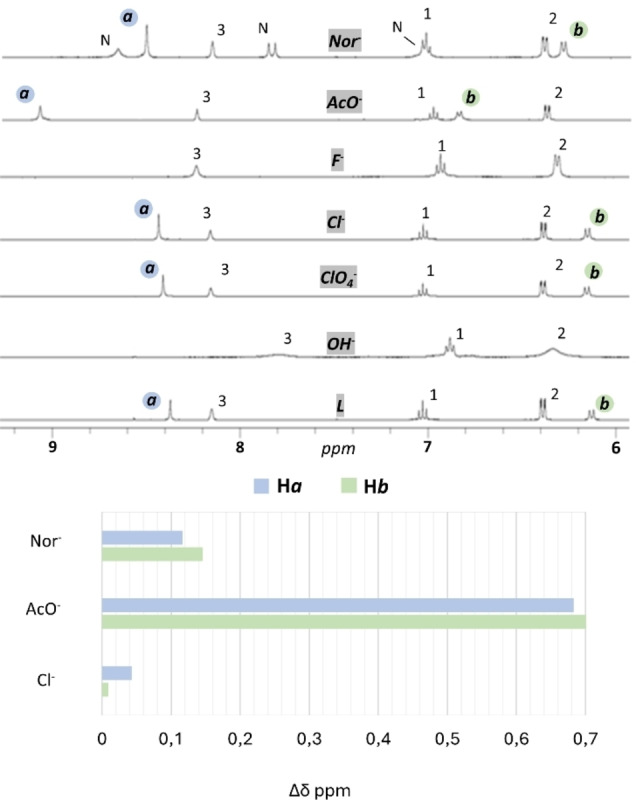
(a) Stacked ^1^H NMR spectra of **L** (7.0 ⋅ 10^−3^ mol L^−1^) recorded in DMSO‐*d*_6_ – 0.5 % D_2_O solution at 298 K after the addition of 3.0 equiv. of G (only 1.0 equiv. of OH^−^ as NMe_4_OH). G (ClO_4_^−^, Cl^−^, F^−^, AcO^−^, Nor^−^ and OH^−^, 0.1 mol L^−1^) were added as tetrabutylammonium salts, except for Nor^−^ that was added as sodium salt, as DMSO‐*d*6 solutions. N are the signals attributed to the resonances of sodium norfloxacin. (b) H*a* and H*b* chemical shift changes upon addition of Cl^−^, AcO^−^ and Nor^−^. See Figure 1 for ^1^H NMR labelling.

The ability of **L** to interact with anions (G) in solution was preliminary tested through a ^1^H NMR screening performed in DMSO‐*d_6_* – 0.5 % D_2_O solution at 298 K by adding 3.0 equiv. of halide anions (F^−^, Cl^−^, Br^−^ and I^−^) or representative oxyanions such as perchlorate (ClO_4_^−^), acetate (AcO^−^) and norfloxacin (Nor^−^). The latter was selected with the aim to test the behaviour of the new receptor towards a more complex carboxylate anion. As shown in Figure 4, significant changes with respect to **L** in ^1^H NMR resonances were observed upon the addition of Cl^−^, F^−^, AcO^−^ and Nor^−^. On the contrary, the addition of 3.0 equiv. of ClO_4_^−^ as well as Br^−^ and I^−^ (spectra not reported) did not induce significant changes in the signals of the ligand (Figure 4), indicating the absence or only a weak interaction with the host.

In the presence of 3.0 equiv. of F^−^ the disappearance of both H*a* and H*b* ureido proton resonances (see Figure 1) together with the broadening of the full spectrum was observed; this is usually seen in similar systems and attributed to a strong interaction between host and guest, suggesting also the deprotonation of the urea fragment.^[45]^ Similarly, the addition of 1.0 equiv. of the strong base NMe_4_OH (reported in Figure 4 as OH^−^), lead to the disappearance of both H*a* and H*b* resonances (see Figure 1), meaning that probably one proton was lost in a deprotonation process occurring at the urea moiety. The latter gave rise to the broadening of the spectrum and to the loss of both H*a* and H*b* signals, thus leading to hypothesize that also the fluoride anion could be sufficiently basic to deprotonate **L**.[^52^, ^53^, ^54^, ^55^, ^56^, ^57^, ^58^, ^59^] The addition of Cl^−^, AcO^−^ and Nor^−^ anions induced an evident perturbation of the spectrum of **L**, in particular of H*a* and H*b*, that persist in the spectra exhibiting the highest shift (Figure 4a–b), suggesting the formation of a −NH⋯G hydrogen bonding occurring at the ureido moiety can be hypothesized. For all guests, the resonances of the tetraazacyclododecane macrocyclic unit remained substantially unchanged, suggesting that, as expected, this portion is not involved in the interaction with the guests (Figure S7).

In order to investigate the **L**‐G interaction and the stability of **L**‐G adducts formed, ^1^H NMR titrations were carried out with all interacting guests (F^−^, Cl^−^, AcO^−^ and Nor^−^).

The addition of an increasing amount of F^−^ (Figure S8) to a DMSO‐*d_6_* ‐ 0.5 % D_2_O solution of **L** caused a gradual downfield shift of the H*a* and H*b* urea resonances. In this case, both signals remained visible until the addition of 0.5 equiv. of F^−^, then after the addition of 1.0 equiv. they broadened up to become hardly detectable upon the addition of 2.0 equiv. of F^−^. The formation of a strong **L**‐F^−^ H‐bonding interaction can be suggested, at least at low F^−^ to **L** molar ratio. However, a concomitant deprotonation process at the ureido moiety probably occurs in the first step of the titration. This is also supported by the appearance of the triplet attributable to the HF_2_^−^ species at around 16.0 ppm, which was visible starting from the addition of 0.5 equiv. of the guest (Figure S8).^[28]^ In any case, the broad spectra obtained did not allow to determine any binding constant through this technique.

The addition of an increasing amount of Cl^−^ (Figure S9) induced a downfield shift of both ureido protons; in particular, H*a* resulted more de‐shielded than H*b* (▵δ=0.06 and 0.02 ppm, respectively, after the addition of 3.0 equiv. of Cl^−^). This could be explained in terms of the difference in the H‐bond strength for this spherical anion, that could be higher with H*a* compared to H*b*, in agreement with the relative distances seen in the solid state, where the hydrogen bond length N‐H*a*⋯Cl^−^ is shorter than N‐H*b*⋯Cl^−^ (N6‐H6N⋯Cl1 2.42 Å *vs*. N5‐H5N⋯Cl1 2.60 Å, respectively, see Table 2). The other resonances were not strongly influenced by the addition of Cl^−^.

Figure S10 reports the spectra of the titration with AcO^−^ in which, by increasing the concentration of G, H*a* and H*b* showed the highest downfield shift among all tested anions (▵δ=0.70 and 0.71 ppm, respectively).

**Figure 5:**
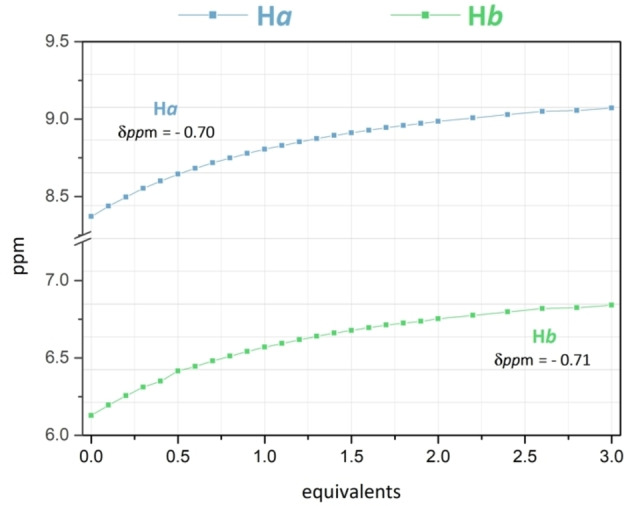
Variation of the H*a* and H*b* chemical shifts as a function of AcO^−^ added (see Figure 1 for ^1^H NMR labelling of **L**), observed during the ^1^H NMR titration of **L** (7.0 ⋅ 10^−3^ mol L^−1^) in DMSO‐*d6* ‐ 0.5 % D_2_O solution at 298 K perfrormed by adding increasing amounts of Bu_4_NAcO (0.1 mol L^−1^) in DMSO‐*d6*.

In addition, the interaction with AcO^−^ perturbed also the resonances of the aromatic protons H1, H2 and H3. After the addition of 3.0 equiv. of AcO^−^, H3 appeared more de‐shielded (▵δ=0.09 ppm) while H1 and H2 appeared more shielded (▵δ=0.05, <0.01 ppm, respectively). The stabilization of the ^1^H NMR resonances occurred only after the addition of more than three equiv. of AcO^−^ (Figure 5 and S10).

**Figure 6:**
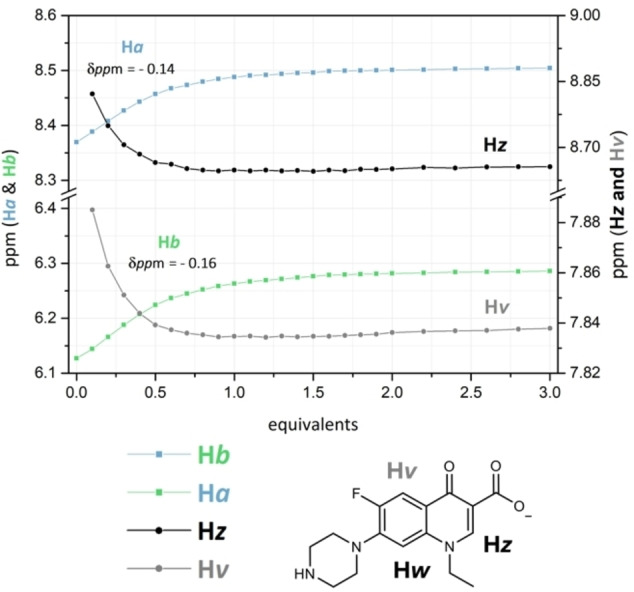
Variation of the resonances of H*a*, H*b*, H*v* and H*z* as a function of Nor^−^ added (See Figure 1 for ^1^H NMR labelling of **L**), observed during the ^1^H NMR titration of **L** (7 ⋅ 10^−3^ mol L^−1^) in DMSO‐*d*6 ‐ 0.5 % D_2_O solution at 298 K performed by adding increasing amounts of sodium norfloxacin (0.1 mol L^−1^) in DMSO‐*d*6.

Finally, the results of the ^1^H NMR titration performed with Nor^−^ (Figure S11) showed the downfield shift of both H*a* and H*b* resonances (▵δ=0.12 and 0.15 ppm, respectively), while those of the phenyl group and Me_2_[12]aneN_4_ unit remained substantially unchanged (Figure 6). The resonances of Nor^−^, H*v* and H*z* exhibited a slight upfield shift, supporting the interaction between **L** and Nor^−^ (Figure 6a‐b). The stack plot reporting the shifts of the urea protons as a function of the increasing amount of the anion (Figure 6) demonstrates that the shift of the signals reached a plateau after the addition of 1 equiv. of Nor^−^ thus suggesting, in agreement with the fitting of the data reported in Figure 6, the formation of a **L**‐Nor^−^ adduct of 1 : 1 stoichiometry. A reverse titration was performed by adding an increasing amount of **L** to a DMSO‐*d*6 – 0.5 % D_2_O solution of Nor^−^ (Figure S12). The data obtained showed that the Nor^−^ signals shifted up to the addition of 1.0 equiv. of **L**, no further shifts of the aromatic signals of the anion were observed at higher **L/**Nor^−^ molar ratio. These results, together with the fitting of the data, suggested only the formation of a complex with a 1 : 1 stoichiometry. In all cases, the ^1^H NMR experiments highlighted that the interaction between **L** and G occurs at the urea functions, *via* the formation of H‐bonding interactions involving G and the ureido NH functions, being these proton signals mainly affected by the presence of the interacting anion.

The ^1^H NMR titrations were processed to evaluate the **L**‐G association constant values (Table 3). In the case of chloride, although the titration highlighted the occurrence of **L–**Cl^−^ interaction, the constant values resulted too low to be safely evaluated by this technique. On the contrary, constant values (log *K*) were determined for AcO^−^ and Nor^−^.

**Table 3:** Logarithms of the association constants of AcO^−^ and Nor^−^ with L (L=**La**, **Lb**, **Lc** and **L**, for AcO^−^; L=**L**, for Nor^−^) in DMSO‐*d*6 – 0.5 % D_2_O solution at 298 K determined by ^1^H NMR titration.

log *K*					
	AcO^−^				Nor^−^
Reaction	La^a^	Lb^a^	Lc^b^	L	L
**L**+G^−^=[**L**G]^−^	2.9(1)^c^	5.6(1)	4.5(1)	2.9(2)	3.6(5)
[**L**G]^−^+G=[**L**G_2_]^2−^	–	2.9(1)	2.1(1)	2.1(4)	–

^a^ From ref [45]; ^b^ from ref [46]; ^c^ values in parentheses are the standard deviation to the last significant figure.

As for the **L**‐AcO^−^ system, the formation of two adducts of 1 : 1 and 2 : 1 AcO^−^/**L** stoichiometry was observed (Table 3), with log *K* values similar for the addition to **L** of the first and second acetate anion to form [**L**AcO]^−^ and [**L**(AcO)_2_]^2−^ species (2.9 and 2.1, respectively). The value for the formation of the [**L**AcO]^−^ species is comparable to that found with the linear parent ligand **La**, while it results lower with respect to those found for the same species with **Lb** and **Lc** (Table 3), where a cooperation of both urea groups in stabilizing the first acetate was suggested. This supports the involvement of only one urea group in binding the acetate anion in **L**. In other words, the two ureido moieties of **L** do not cooperatively act in binding AcO^−^ and, probably, the interaction between **L** and AcO^−^ takes place outside the macrocyclic cavity, involving only one urea group, in a similar way as shown in Figure S5 for **7**.

On the contrary, upon the addition of norfloxacin anion only the formation of the [**L**Nor]^−^ species with log *K*=3.6 (Table 3) was observed.

In this case, it is more difficult to suggest an inward/outward interaction between **L** and Nor^−^, due to the higher log *K* value with respect to acetate and the absence of a crystal structure with [**L**Nor]^−^ adduct.

**Figure 7:**
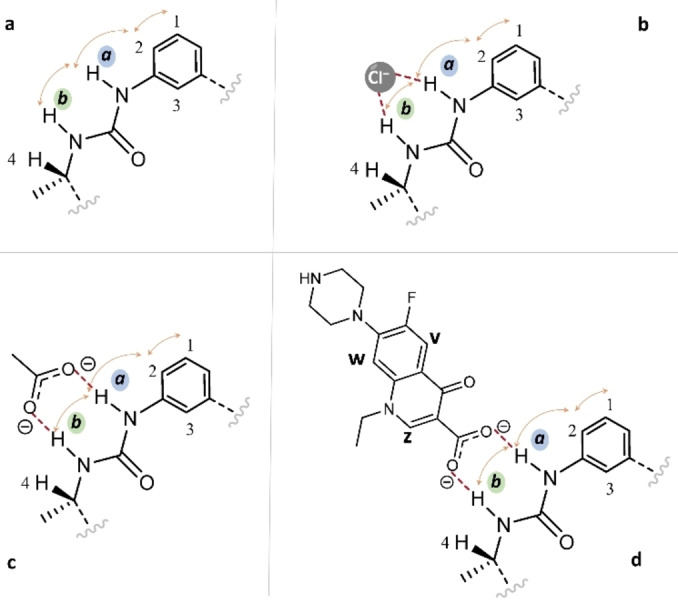
Spatial proximity observed from ^1^H NOESY (orange arrows) and proposed interaction mechanisms between **L** (a) and G (G=Cl^−^ (b), AcO^−^ (c) and Nor^−^ (d)).

Trying to better clarify this aspect, Nuclear Overhauser Effect (NOE) NMR studies were performed to understand the conformation of the free hosting molecule and its possible changes upon the anion binding. As represented in Figure 7a, the NOE effects observed between H1↔H2, H2↔H*a* and H*a*↔H*b* in the free form of **L** suggest for the ligand in solution a conformation where both −NH functions point outside the cavity (Figure S13).

The results of the same NOE experiments performed after the addition of 1.0 equiv. of Cl^−^, AcO^−^ or Nor^−^ to a solution of **L** show the same spatial proximity observed in the free **L**, with no additional NOE effect (Figures 7b–c and S14–S16). These results point at an outward interaction of **L** with all guests (Figure 7), therefore in a way similar to that found in the three crystal structures of H**L** ^+^ species (**7**–**9**) herein reported.

## Conclusions

A new macro‐bicyclic receptor (**L**) for anions was synthesized and characterized. **L** shows a cage topology based on a tetraaza‐macrocyclic scaffold bearing a long chain connected through the *trans* nitrogen atoms of the macrocycle and containing two urea groups spaced by a phenyl bridge. The macrocyclization was obtained in good yield by high‐dilution technique forming the two urea functionalities between bis‐isocyanate and chiral free diamine **5**. The latter was easily prepared by double amidation of the pentafluorophenol active esters of N‐Cbz L‐alanine with bis‐secondary amine 1,7‐dimethyl‐1,4,7,10‐tetraazacyclododecane followed by double removal of the protecting group by catalytic hydrogenolysis.

The results of crystal structures analysis and ^1^H NMR solution studies performed in DMSO‐*d6* ‐ 0.5 % D_2_O solution suggest the ability of **L** to interact with several anions such as AcO^−^, Nor^−^ and Cl^−^ through H‐bonding involving the urea functions. Association constants for the formation of [**L**G]^−^ species with AcO^−^ and Nor^−^ were determined by NMR data; the values retrieved with the bifurcated carboxylate anions ([**L**AcO]^−^ log *K*=2.9; [**L**Nor]^−^ log *K*=3.6) are higher than that found for the spherical Cl^−^ (log *K* <1). These values are lower compared to the binding constant values found for the macrocyclic **Lb** and **Lc** ligands in the previous studies (log *K*=5.6 for [**Lb**AcO]^−^ and 4.5 for [**Lc**AcO]^−^), where the two urea functions cooperate in the binding of acetate,[^51^, ^52^] but they are similar to that found for the linear mono‐urea‐based ligand **La** (log *K*=2.9 for [**La**AcO]).^[51]^

These results suggest that **L** binds in solution the guest anions *via* H‐bonding involving only one urea group, probably pointing outward the macrocyclic cavity.

This is also supported by ^1^H NOESY experiments. In conclusion, the higher preorganisation of **L** provided by the connection of the two side arms through an aromatic spacer to form a cage, with respect to the previously studied parent ligands **Lb** and **Lc**, seems to prevent the ability of the urea groups to cooperate in anion binding, converging inward the cavity. Taking advantage from these results, future developments will focus on the variation of the cavity size as well as the functionalization of the aromatic spacer in order to: i) enhance the solubility in water; ii) allow for the cooperation of the two binding groups inside the macrocyclic cavity; iii) provide the receptor with optical properties that would allow for the signalling of the occurred interaction and would widen the range of applications of the receptor (*e. g*. catalysis, molecular recognition, etc).

## Experimental Section

### Materials and Methods

All reactions were run in air unless otherwise noted. Column chromatography purifications were performed in flash chromatography conditions using 230–400 mesh silica gel. Analytical thin layer chromatography (TLC) was carried out on silica gel plates (Silica Gel 60 F254) that were visualized by exposure to ultraviolet light. ^1^H‐NMR (400 MHz) and ^13^C‐NMR (100 MHz) spectra were determined on Bruker instruments equipped with a variable temperature controller. The temperature of the NMR probe was calibrated using 1,2‐ethandiol as calibration sample. NMR spectra were recorded at 298 K, chemical shifts (δ scale) for ^1^H‐NMR and ^13^C‐NMR were reported in parts per million (ppm values), referenced relative to residual proton in the deuterated solvent, with coupling constants (*J* values) reported in hertz (Hz). ^1^H‐^1^H and ^1^H‐^13^C correlation experiments were performed using standard Bruker pulse sequence to assign the signals. The 2D‐Noesy experiments were conducted using a delay (*d1*) of 2.0 s and a mixing time (*d8*) of 0.40 s. Optical rotation analysis was performed with a polarimeter using a sodium lamp (λ=589 nm, D‐line); [α]_D_^20^ values are reported in 10^−1^ deg cm^2^ g^−1^; concentration (c) is in g for 100 mL. HRMS analysis was performed using Orbitrap Exploris mass spectrometers.

### Synthesis

Benzyloxycarbonyl‐L‐Alanine (**1**) and 1‐cyanato‐3‐isocyanatobenzene (**6**) are commercially available. 1,7‐dimethyl‐1,4,7,10‐tetra‐zacyclododecane (**3**) was synthesized according to the literature procedure.^[60]^

**Dibenzyl((2R,2′R)‐(4,10‐dimethyl‐1,4,7,10‐tetraazacyclododecane‐1,7‐diyl)bis(1‐oxopropane‐2,1‐diyl))dicarbamate (4)**. Pyridine (5.2 mmol, 0.42 mL) and pentafluorophenyl‐2,2,2‐trifluoroacetate (5.6 mmol, 0.96 mL) were added to a solution of Cbz‐L‐Alanine **1** (4.3 mmol, 0.96 g) in DMF (4.3 mL, 1 M). The reaction mixture was stirred for 1 hour at room temperature, diluted with AcOEt (150 mL) and washed with 0.1 M aqueous HCl (3x25 mL), 5 % aqueous NaHCO_3_ (3x25 ml) and brine (25 mL). The organic solution was dried over Na_2_SO_4_ and concentrated under reduced pressure to give the desiderate pentafluorophenyl ester **2** in almost quantitative yield. A solution of the above pentafluorophenyl ester **2** (4.2 mmol, 1.63 g) in dry DMF (2,5 mL) was slowly added dropwise over 30 min at 0 °C to a stirred solution of 1,7‐dimethyl‐1,4,7,10‐tetraazacyclododecane **3** (1.5 mmol, 0.3 g) and i‐Pr_2_EtN (6.6 mmol, 1.15 mL) in dry DMF (1 mL). The reaction mixture was stirred for 72 hours at room temperature. The organic solution was concentrated under reduced pressure and the residue was purified by silica gel chromatography to give intermediate **4** (0.64 g). Yield 67 %. Rf 0.3 (9 : 1; CH_2_Cl_2_:MeOH). MS (ESI): 611.3 [M+H]^+^. ^1^H‐NMR (CDCl_3_): δ 0.95 (d, 6H, *J*=4 Hz), 2.28 (s, 6H), 2.24‐2.56 (m, 8H), 3.41‐3.56 (m, 8H), 4.48 (bs, 2H), 5.09 (s, 4H), 6.64 (bs, 2H), 7.34 (s, 10H). ^13^C‐NMR (CDCl_3_): δ 18.7, 43.5, 45.4, 55.3, 57.7, 67.0, 127.9, 128.2, 128.5, 136.5, 155.5, 171.1. [α]_D_^20^=−55.4 (c=0.80, CHCl_3_).

**2R,2′R)‐1,1′‐(4,10‐dimethyl‐1,4,7,10‐tetraazacyclododecane‐1,7‐diyl)bis(2‐aminopropan‐1‐one (5)**. 10 % Pd/C (1.7 mmol, 182 mg) was added to a solution of intermediate **4** (0.99 mmol, 0.42 g) in EtOH abs (6.9 mL, 0.1 M). The mixture was stirred under 1 atm of H_2_ for 16 hours at room temperature, then filtered through Celite and the solvent was removed under vacuum to give the desired diamine in quantitative yield that was used in the next step without any further purification.

**(3S,15S)‐3,15,20,25‐tetramethyl‐1,4,6,12,14,17,20,25‐octaazatricyclo[15.5.5.17.11]octacosa‐7(28),10‐diene‐2,5,13,16‐tetraone (L)**. A solution of **5** (1.257 g, 3.67 mmol) in MeOH (160 mL) and a solution of **6** (0.58 g, 3.67 mmol) in dry CH_2_Cl_2_ (160 mL) were very slowly added simultaneously dropwise over 1 hour to a stirred solution of dry CH_2_Cl_2_ (60 mL) and MeOH (60 mL). The reaction mixture was stirred for 72 hours at room temperature. The solution was concentrated under reduced pressure and the residue was purified by silica gel chromatography (70‐230 mesh) to give **L** (0.85 g). Yield 46 %. Rf 0.25 (8 : 2; CH_3_CN : NH_4_OH). MS (ESI): 503.42 [M+H]^+^. ^1^H NMR (400 MHz, DMSO‐*d_6_*, 25 °C): *δ*=1.14 (d, 6H, *J*=7.0 Hz), 2.28 (s, 6H), 2.77 (m, 8H), 3.67 (m, 2H), 3.82 (m, 2H), 4.54 (m, 2H), 6.14 (d, 2H, *J*=8.4 Hz), 6.39 (dd, 2H, *J*=7.9, *J*=2.1 Hz), 7.03 (t, 1H, *J*=7.9 Hz), 8.15 (s, 1H), 8.39 (s, 2H) ppm (Figure S17 ^1^H NMR spectrum). ^13^C NMR (100 MHz, DMSO‐*d_6_*, 25 °C): *δ*=17.28, 46.55, 47.25, 48.64, 56.38, 113.24, 114.44, 128.68, 140.38, 156.22, 173.99 ppm (Figure S18 ^13^C NMR spectrum). [α]_D_^20^=98.47 (c=0.0021, CH_3_COOH).

### Synthesis of the Crystals

**HL**⋅**AcO (7)**. A freshly prepared solution of TBAAcO in methanol (2.41 mg, 0.008 mmol, 0.5 mL) was added to solution of **L** in methanol (2 mg, 0.004 mmol, 5 mL). The mixture was kept at RT and after slow evaporation of the solvent (3 days) it has been observed the formation of a precipitate as colourless needles suitable for X‐ray analysis.

**HL**⋅**ClO_4_ (8)**. A freshly prepared solution of TBAClO_4_ in methanol (2.72 mg, 0.008 mmol, 0.5 mL) was added to solution of **L** in methanol (2 mg, 0.004 mmol, 5 mL). The mixture was kept at RT and after slow evaporation of the solvent (3 days) it has been observed the formation of a precipitate as colourless needles suitable for X‐ray analysis.

**HL**⋅**Cl (9)**. A freshly prepared solution of TBACl in methanol (2.22 mg, 0.008 mmol, 0.5 mL) was added to solution of **L** in methanol (2 mg, 0.004 mmol, 5 mL). The mixture was kept at RT and after slow evaporation of the solvent (3 days) it has been observed the formation of a precipitate as colourless needles suitable for X‐ray analysis.

### Single Crystal X‐Ray Diffraction

Single crystal X‐ray diffraction data of H**L**⋅AcO⋅2.5H_2_O (**7**) and H**L**⋅Cl⋅2.5H_2_O (**9**) were collected, at 150 K, on an Oxford Diffraction Xcalibur diffractometer equipped with a CCD area detector, using Mo−Kα radiation (0.71073 Å), monochromated with a graphite prism. Data were collected and reduced through the CrysAlisPro program.[^61^, ^62^] Absorption correction was performed with the ABSPACK program in CrysAlisPro.

Single crystal data for H**L**⋅ClO_4_⋅H_2_O⋅MeOH (**8**), were collected, at 110 K, on a Bruker Apex‐II diffractometer equipped with a CCD detector, controlled using APEX2 software, using Cu−Kα radiation (λ=1.54184 Å).^[63]^ An Oxford Cryostream was used to control the temperature of the crystals prior to data collection. Data integration and reduction were performed using the Bruker SAINT software.^[64]^

The crystal structures were solved using the SIR‐2004 package^[65]^ and refined by full‐matrix least squares against *F* ^*2*^ using all data (SHELXL‐2018/3).^[66]^ In all the three structures, all the non‐hydrogen atoms, with the exception of the oxygen atoms of the disordered perchlorate anion in **8**, were refined with anisotropic displacement parameters. Concerning the hydrogen atoms, in **8** they were all found in the Fourier Density Map and their positions were freely refined while their thermal parameter was set in accordance with the atom to which they are bonded. In **7** and **9** all the hydrogen atoms bonded to nitrogen or oxygen atoms were found in the Fourier Density Map and their positions were freely refined while their thermal parameter was set in accordance with the atom to which they are bonded, while those bonded to carbon atoms were set in calculated positions. Finally, the perchlorate anion in **8** was disordered, such disorder was modelled by introducing on the refinement three positions for each oxygen atom, the occupancy factors for the three models are: 0.282, 0.395 and 0.323.

Geometrical calculations were performed by PARST97^[67]^ and molecular plots were produced by the program Mercury (v2024.1.0)^[68]^ and Discovery Studio Visualizer 2019.^[69]^

Deposition Numbers 2385625 (for **7**), 2385627 (for **8**), 2385626 for (**9**) contain the supplementary crystallographic data for this paper. These data are provided free of charge by the joint Cambridge Crystallographic Data Centre and Fachinformationszentrum Karlsruhe Access Structures service.

### NMR Experiments

In the binding studies all G are tetrabutylammonium salts except for Nor^−^ which has been added as sodium salt. ^1^H NMR spectra of the preliminary screening experiment of **L** with anions (Figure 4) were acquired in DMSO‐*d6* ‐ 0.5 % D_2_O solvent mixture after the addition of 3.0 equiv. of G or 1.0 equiv. of NMe_4_OH to 7.0 ⋅ 10^−3^ mol L^−1^ solutions of the ligand. In the titration experiments, AcO^−^, Cl^−^ and Nor^−^ anions were added 0.1 equiv. at a time as tetrabutylammonium salts dissolved in DMSO‐*d6* by direct addition to the NMR tube; the NMR tube was kept for 5 min at a temperature of 298.0 K before starting the acquisition of each spectrum. The small amount of D_2_O (0.5 %) was added to avoid the uncontrolled absorption of water by DMSO‐*d*6 during the measurements, keeping a fixed amount of water in solution.

The association constants were calculated by using the HYP NMR computer program.[^70^, ^71^]

## Conflict of Interests

There are no conflicts to declare.

1

## Supporting information

As a service to our authors and readers, this journal provides supporting information supplied by the authors. Such materials are peer reviewed and may be re‐organized for online delivery, but are not copy‐edited or typeset. Technical support issues arising from supporting information (other than missing files) should be addressed to the authors.

Supporting Information

## Data Availability

The data that support the findings of this study are available from the corresponding author upon reasonable request.

## References

[1] P. A. Gale , N. Busschaert , C. J. E. Haynes , L. E. Karagiannidis , I. L. Kirby , Chem. Soc. Rev. 2014, 43, 205–241. 24108306 10.1039/c3cs60316d

[2] N. Busschaert , C. Caltagirone , W. V. Roosom , P. A. Gale , Chem. Rev. 2015, 115, 8038–8155. 25996028 10.1021/acs.chemrev.5b00099

[3] M. J. Langton , C. J. Serpell , P. D. Beer , Angew. Chem. Int. Ed. 2016, 55, 1974–1987. 10.1002/anie.201506589 PMC4755225 26612067

[4] P. A. Gale , E. N. W. Howe , X. Wu , Chem 2016, 1, 351–422.

[5] N. H. Evans , P. D. Beer , Angew. Chem. Int. Ed. 2014, 53, 11716–11754. 10.1002/anie.201309937 25204549

[6] G. Picci , R. Montis , A. M. Gilchrist , P. A. Gale , C. Caltagirone , Coord. Chem. Rev. 2024, 501, 215561.

[7] G. Picci , R. Montis , V. Lippolis , C. Caltagirone , Chem. Soc. Rev. 2024, 53, 3952–3975. 38465875 10.1039/d3cs01165h

[8] P. D. Beer , P. A. Gale , Angew. Chem. Int. Ed. 2001, 40, 486–516. 11180358

[9] R. Martínez-Máñez , F. Sancenón , Chem. Rev. 2003, 11, 4419–4476. 10.1021/cr010421e 14611267

[10] K. Brak , E. N. Jacobsen , Angew. Chem. Int. Ed. 2013, 52, 534–561. 10.1002/anie.201205449 PMC4284951 23192886

[11] A. P. Davis , D. N. Sheppard , B. D. Smith , Chem. Soc. Rev. 2007, 36, 348–357. 17264935 10.1039/b512651g PMC2854546

[12] X. Wu , L. K. Macreadie , P. A. Gale , Coord. Chem. Rev. 2021, 432, 213708.

[13] M. Formica , V. Fusi , D. Paderni , G. Ambrosi , M. Inclan , M. P. Clares B Verdejo , E. García-España , Molecules 2021, 26, 2352. 33919489 10.3390/molecules26082352 PMC8073790

[14] E. Macedi , L. Giorgi , M. Formica , P. Rossi , D. Paderni , P. Paoli , V. Fusi , ChemPlusChem 2023, 88, e202200364. 36658696 10.1002/cplu.202200364

[15] J. M. Llinares , D. Powell , K. Bowman-James , Coord. Chem. Rev. 2003, 240, 57–75.

[16] C. Bazzicalupi , A. Bencini , C. Giorgi , B. Valtancoli , V. Lippolis , A. Perra , Inorg. Chem. 2011, 50, 7202–7216. 21710997 10.1021/ic2007815

[17] A. Bencini , C. Coluccini , A. Garau , C. Giorgi , V. Lippolis , L. Messori , D. Pasini , S. Puccioni , Chem. Commun. 2012, 48, 10428–10430. 10.1039/c2cc35383k 22983504

[18] N. Kaur , G. Kaur , U. A. Fegade , A. Singh , S. K. Sahoo , A. S. Kuwar , N. Singh , Trends Anal. Chem. 2017, 95, 86–109.

[19] J. Zhao , D. Yang , X. J. Yang , B. Wu , Coord. Chem. Rev. 2019, 378, 415–444.

[20] V. Amendola , L. Fabbrizzi , L. Mosca , Chem. Soc. Rev. 2010, 39, 3889–3915. 20818452 10.1039/b822552b

[21] V. Amendola , M. Boiocchi , B. Colasson , L. Fabbrizzi , Inorg. Chem. 2006, 45, 6138–6147. 16878922 10.1021/ic060160x

[22] F. C. Parks , E. G. Sheetz , S. R. Stutsman , A. Lutolli , S. Debnath , K. Raghavachari , A. H. Flood , J. Am. Chem. Soc. 2022, 144, 1274–1287. 35015538 10.1021/jacs.1c10758

[23] M. P. Teulade-Fichou , J. P. Vigneron , J. M. Lehn , J. Chem. Soc. Perkin Trans. 2 1996, 2169–2175.

[24] L. Fabbrizzi , M. Licchelli , G. Rabaioli , A. Taglietti , Coord. Chem. Rev. 2000, 205, 85–108.

[25] L. Adriaenssens , P. Ballester , Chem. Soc. Rev. 2013, 42, 3261–3277. 23321897 10.1039/c2cs35461f

[26] L. Chen , S. N. Berry , X. Wu , E. N. W. Howe , P. A. Gale , Chem. 2020, 6, 61–141.

[27] Y. Chen , G. Wu , L. Chen , L. Tong , Y. Lei , L. Shen , T. Jiao , H. Li , Org. Lett. 2020, 22, 4878–4882. 32496778 10.1021/acs.orglett.0c01722

[28] S. O. Kang , J. M. Llinares , D. Powell , D. VanderVelde , K. Bowman-James , J. Am. Chem. Soc. 2003, 125, 10152–10153. 12926920 10.1021/ja034969+

[29] H. Xie , V. W. Liyana Gunawardana , T. J. Finnegan , W. Xie , J. D. Badjić , Angew. Chem. Int. Ed. 2022, 61, e202116518 (1–7). 10.1002/anie.202116518 35038355

[30] L. Jing , E. Deplazes , J. K. Clegg , X. Wu , Nat. Chem. 2024, 16, 335–342. 38351381 10.1038/s41557-024-01457-5

[31] D. A. McNaughton , W. G. Ryder , A. M. Gilchrist , P. Wang , M. Fares , X. Wu , P. A. Gale , Chem 2023, 9, 3045–3112.

[32] S. K. Kim , J. Lee , N. J. Williams , V. M. Lynch , B. P. Hay , B. A. Moyer , J. L. Sessler , J. Am. Chem. Soc. 2014, 136, 15079–15085. 25254498 10.1021/ja5086996

[33] J. Bartl , S. Kubik , ChemPlusChem 2020, 85, 963–969. 32406613 10.1002/cplu.202000255

[34] J. M. Lehn , Supramolecular Chemistry. Concepts and Perspectives, Wiley-VCH Verlag Gmbh, Weinheim, 1995.

[35] J. L. Sessler , P. A. Gale , V. S. Cho , Anion Recognition Chemistry (Eds.: J. F. Stoddart ), Royal Society of Chemistry, Cambridge, 2006.

[36] M. W. Hosseini , J. M. Lehn , J. Am. Chem. 1982, 104, 3525–3527.

[37] R. Mobili , G. Preda , S. La Cognata , L. Toma , D. Pasini , V. Amendola , Chem. Commun. 2022, 58, 3897–3900. 10.1039/d2cc00612j 35234783

[38] V. McKee , J. Nelson , R. M. Town , Chem. Soc. Rev. 2003, 32, 309–325. 14518184 10.1039/b200672n

[39] G. G. Morgan , V. Mckee , J. Nelson , J. Chem. Soc. Chem. Commun. 1995, 16, 1649–1652.

[40] M. W. Hosseini , J. M. Lehn , Helv. Chim. Acta 1998, 71, 749–756.

[41] A. Bianchi , K. Bowman-James , E. García-España , Anion Coordination chemistry, Wiley-VCH: Weinheim, 2011.

[42] K. Bowman-James , Acc. Chem. Res. 2005, 38, 671–678. 16104690 10.1021/ar040071t

[43] J. W. Steed , Chem. Soc. Rev. 2009, 38, 506–519. 19169464 10.1039/b810364j

[44] G. Montà-González , F. Sancenón , R. Martínez-Máñez , V. Martí-Centelles , Chem. Rev. 2022, 122, 13636–13708. 35867555 10.1021/acs.chemrev.2c00198 PMC9413269

[45] M. Formica , V. Fusi , E. Macedi , P. Paoli , G. Piersanti , P. Rossi , G. Zappia , P. Orlando , New J. Chem. 2008, 32, 1204–1214.

[46] M. Formica , V. Fusi , L. Giorgi , E. Macedi , G. Piersanti , M. A. Varrese , G. Zappia , Supramol. Chem. 2010, 22, 365–379.

[47] P. Knops , N. Sendhoff , H. B. Mekelburger , F. Vogtle , Top. Curr. Chem. 1992, 161, 1–36.

[48] D. H. Busch , J. Inclusion Phenom. Macrocyclic Chem. 1992, 12, 389.395.

[49] M. Retini , F. Bartoccini , G. Zappia , G. Piersanti , Eur. J. Org. Chem. 2021, 5, 825–829.

[50] Stereochemical and Stereophysical Behaviour of Macrocycles (Ed. I. Bernal ), Elsevier, Amsterdam, 1987, p.34.

[51] G. R. Desiraju , Angew. Chem. Int. Ed. 2011, 50, 52–59. 10.1002/anie.201002960 21031379

[52] T. Gunnlaugsson , H. D. P. Ali , M. Glynn , P. E. Kruger , G. M. Hussey , F. M. Pfeffer , C. M. G. dos Santos , J. Tierney , J. Fluoresc. 2005, 15, 287–299. 15986154 10.1007/s10895-005-2627-y

[53] T. Gunnlaugsson , A. P. Davis , J. E. O′Briena , Mark Glynn , Org. Biomol. Chem. 2005, 3, 48–56. 15602598 10.1039/b409018g

[54] F. M. Pfeffer , A. M. Buschgens , N. W. Barnett , T. Gunnlaugsson , P. E. Kruger , Tetrahedron Lett. 2005, 46, 6579–6584.

[55] F. M. Pfeffer , T. Gunnlaugsson , P. Jensen , P. E. Kruger , Org. Lett. 2005, 7, 5357–5360. 16288505 10.1021/ol051497q

[56] T. Gunnlaugsson , P. E. Kruger , P. Jensen , F. M. Pfeffer , G. M. Hussey , Tetrahedron Lett. 2003, 44, 8909–8913.

[57] S. Camiolo , P. A. Gale , M. B. Hursthouse , M. E. Light , Org. Biomol. Chem. 2003, 1, 741–744. 12929465 10.1039/b210848h

[58] D. E. Gomez , L. Fabbrizzi , M. Licchelli , E. Monzani , Org. Biomol. Chem. 2005, 3, 1495–1500. 15827647 10.1039/b500123d

[59] R. Montis , A. Bencini , S. J. Coles , L. Conti , L. Fusaro , P. A. Gale , C. Giorgi , P. N. Horton , V. Lippolis , L. K. Mapp , C. Caltagirone , Chem. Commun. 2019, 55, 2745–2748. 10.1039/c8cc09962f 30676586

[60] G. Piersanti , M. A. Varrese , V. Fusi , L. Giorgi , G. Zappia , Tetrahedron Lett. 2012, 51, 3436–3438.

[61] CrysAlisPro, version 1.171.34.41; Oxford Diffraction Ltd. (release 13–09-**2010** CrysAlis171.NET).

[62] CrysAlisPro, version 1.171.35.19; Oxford Diffraction Ltd. (release 27–10-**2011** CrysAlis171.NET).

[63] Bruker (**2012**). Bruker APEX2. Bruker AXS Inc., Madison, Wisconsin, USA.

[64] Bruker (**2012**). Bruker SAINT. Bruker AXS Inc., Madison, Wisconsin, USA.

[65] M. C. Burla , R. Caliandro , M. Camalli , B. Carrozzini , G. L. Cascarano , L. Da Caro , C. Giacovazzo , G. Polidori , R. Spagna , J. Appl. Crystallogr. 2005, 38, 381–388.

[66] G. M. Sheldrick , Acta Crystallogr. Sect. C 2015, 71, 3–8. 10.1107/S2053273314026370 PMC4283466 25537383

[67] M. Nardelli , J. Appl. Crystallogr. 1995, 28, 659.

[68] C. F. Macrae , I. Sovago , S. J. Cottrell , P. T. A. Galek , P. McCabe , E. Pidcock , M. Platings , G. P. Shields , J. S. Stevens , M. Towler , P. A. Wood , J. Appl. Crystallogr. 2020, 53, 226–235. 32047413 10.1107/S1600576719014092 PMC6998782

[69] Dassault Systèmes BIOVIA, Discovery Visualizer, v19.1.0.18287 (**2019**), San Diego: Dassault Systèmes.

[70] C. Frassinetti , S. Gelli , A. Sabatini , M. S. Moruzzi , A. Vacca , Anal. Biochem. 1995, 231, 375. 10.1006/abio.1995.9984 8594988

[71] P. Gans , A. Sabatini , A. Vacca , Talanta 1996, 43, 1739–1753. 18966661 10.1016/0039-9140(96)01958-3

