# Supplementary material for: A New Bis‐Urea Based Cage Receptor for Anions: Synthesis, Solid State Structures and Binding Studies
Source: Chem Asian J. 2024 Dec 6;20(2):e202401258. doi: 10.1002/asia.202401258 (PMC11741156; doi:10.1002/asia.202401258)
Supplement: Supplementary file 1 — Supporting Information [file ASIA-20-e202401258-s001.pdf]

// Chemistry - An Asian Journal

Supporting Information

A New Bis-Urea Based Cage Receptor for Anions: Synthesis, Solid State Structures and Binding Studies

Daniele Paderni,* Mauro Formica,* Eleonora Macedi, Luca Giorgi, Patrizia Rossi, Michele Retini, Nicola De Cata, Giovanni Zappia, Giovanni Piersanti, and Vieri Fusi*

Supporting Information

A New Bis-Urea Based Cage Receptor for Anions: Synthesis, Solid State Structures and Binding Studies

Daniele Paderni,[a] Mauro Formica,*[a] Eleonora Macedi,[a] Luca Giorgi,[a] Patrizia Rossi,[b] Michele Retini,[c] Nicola De Cata,[c] Giovanni Zappia,[d] Giovanni Piersanti,[c] and Vieri Fusi*[a]*

[a] Department of Pure and Applied Sciences, University of Urbino, via Ca' le Suore 2-4, 61029, Urbino, Italy.

E-mail: daniele.paderni@uniurb.it, mauro.formica@uniurb.it, vieri.fusi@uniurb.it

[b] Department of Industrial Engineering, University of Firenze, via Santa Marta 3, 50139, Firenze, Italy

[c] Department of Biomolecular Sciences, University of Urbino, via Ca' le Suore 2-4, 61029, Urbino, Italy

[d] Dipartimento di Promozione delle Scienze Umane e della Qualità della Vita, University San Raffaele, via di Val Cannuta, 247, Rome, Italy

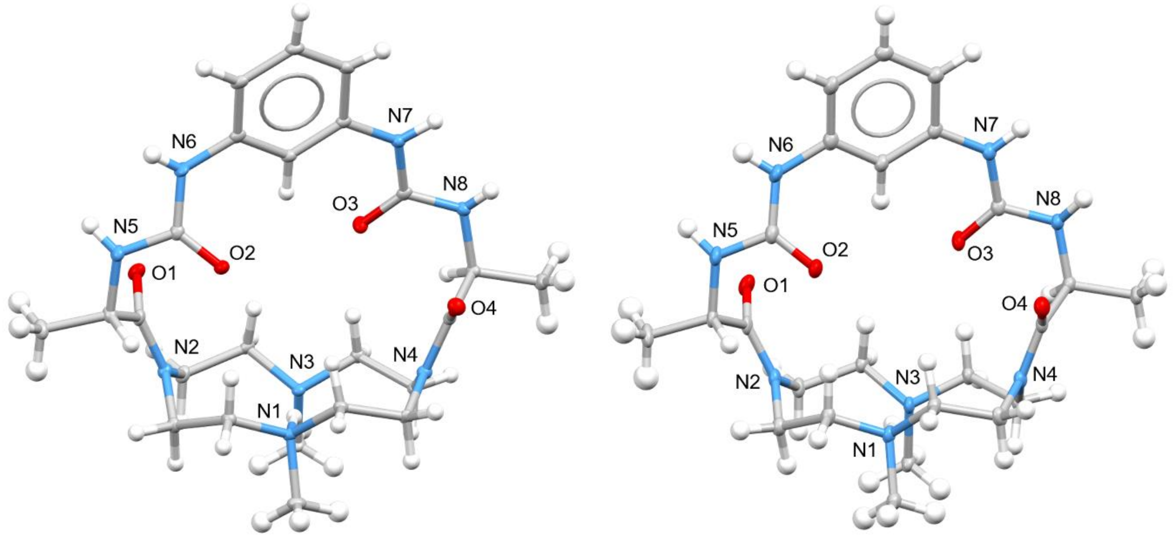

Figure S1. ORTEP view of the H**L**$^+$ cation in **8** (left) and **9** (right). Ellipsoid probability = 30%.

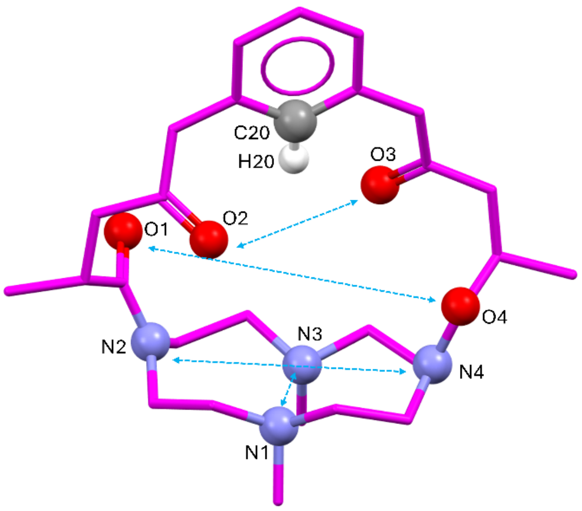

Figure S2. Distances defining the macrocyclic cavity dimensions.

Table S1. Distances defining the cavity of the ligand cation in the three structures **7**, **8** and **9**.

**Distances (Å)**	**7**	**8**	**9**
N1···N3	2.753(5)	2.748(4)	2.715(6)
N2···N4	5.596(5)	5.622(4)	5.563(5)
O1···O4	7.391(5)	7.351(4)	7.280(5)
O2···O3	3.978(4)	3.774(4)	3.786(4)
H20···(N1, N2, N3, N4) mean plane	4.5692(1)	4.58(5)	4.5362(1)

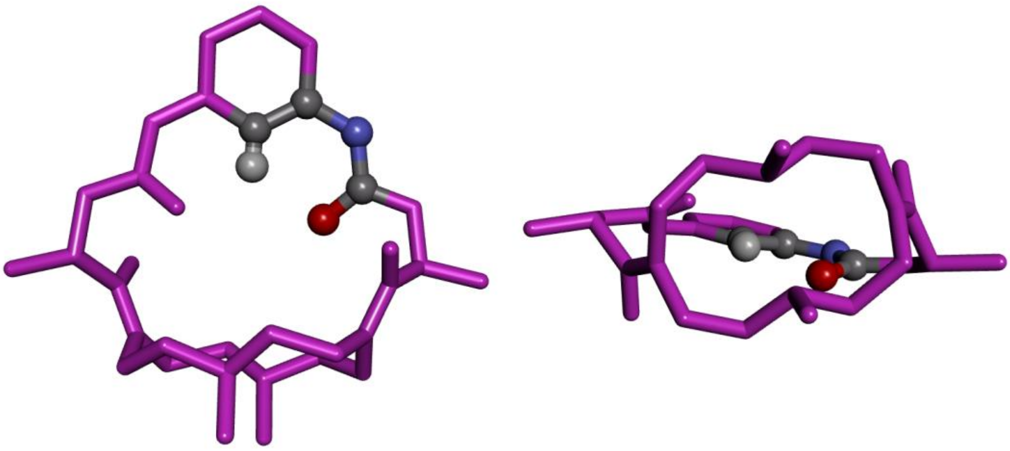

Figure S3. Six members ring (ball and stick representation) due to the C-H···O=C H-bond interaction in H**L**$^{+}$.

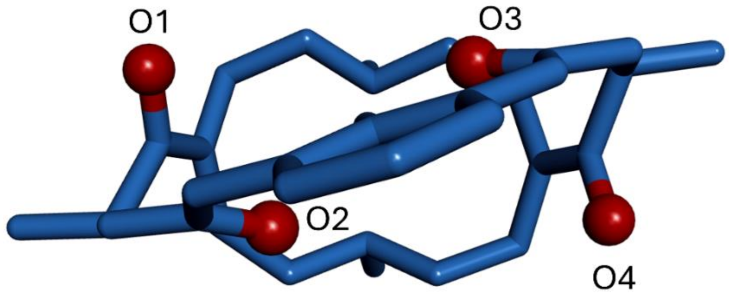

Figure S4. C=O disposition in H**L**$^{+}$. Oxygen atoms in red ball and stick style.

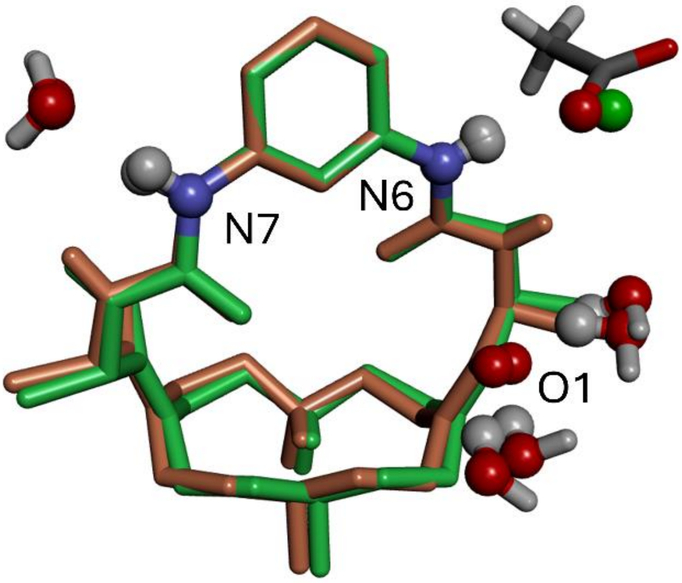

Figure S5. Intermolecular H-bond interactions in **7** (green) and **9** (orange). Atoms involved in the interaction are reported in ball and stick style.

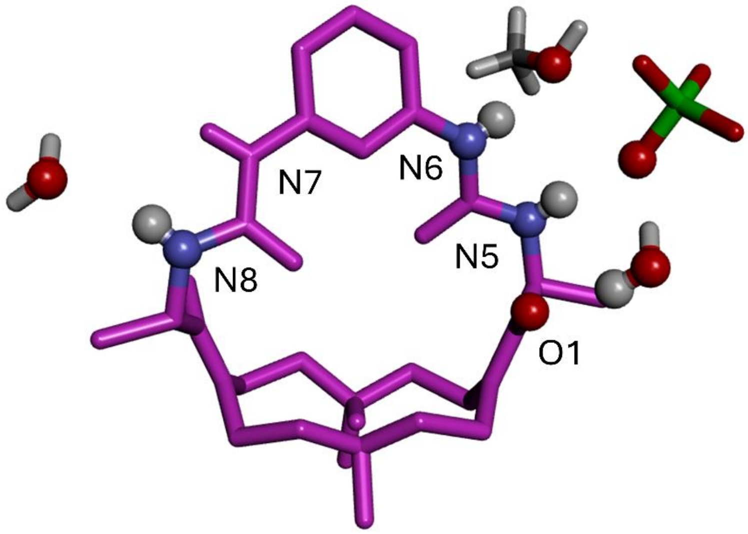

Figure S6. Intermolecular H-bond interactions in **8**. Atoms involved in the interaction are reported in ball and stick style.

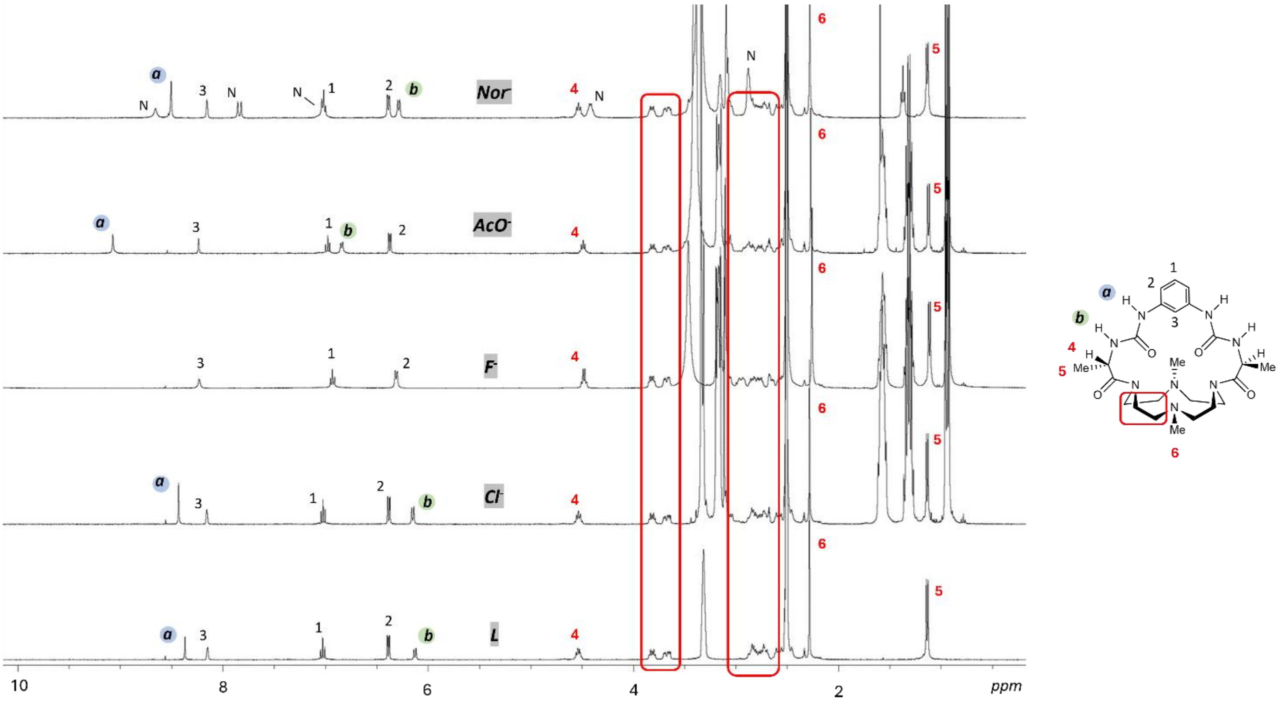

Figure S7. Stacked ^{1}H NMR spectra of **L** ($7.0 \cdot 10^{-3}$ mol·L^{-1}) recorded in DMSO-d_6 – 0.5% D_2O solution at 298 K after the addition of 3.0 equiv of the interacting G. G (0.1 mol·L^{-1}) were added as tetrabutylammonium salts DMSO-d_6 solutions, except for Nor$^-$ that was added as sodium salt. N are the signals attributed to the resonances of sodium norfloxacin.

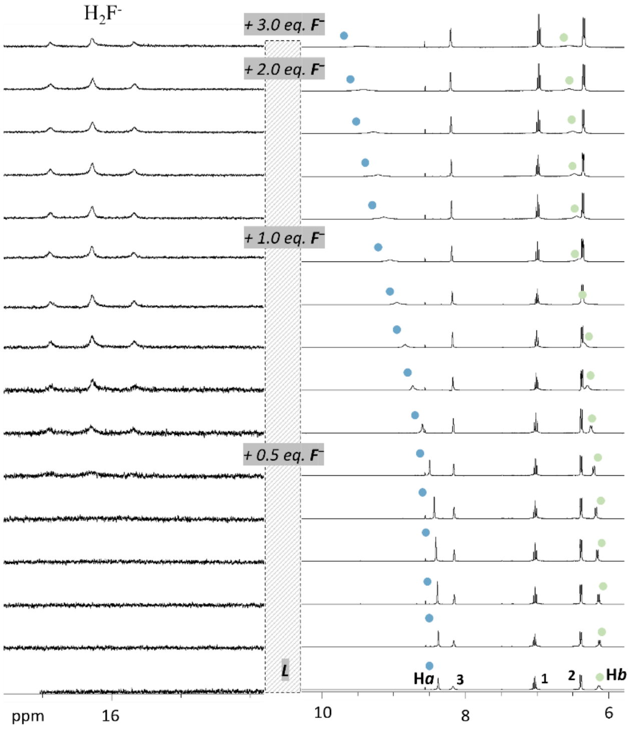

Figure S8. ^{1}H NMR titration of **L** ($7.0 \cdot 10^{-3}$ mol·L^{-1}) recorded in DMSO-d_6 - 0.5% D_2O solution at 298 K by adding increasing amounts of Bu_4NF (0.1 mol·L^{-1}) in DMSO-d_6. See Figure S7 for ^{1}H NMR labelling.

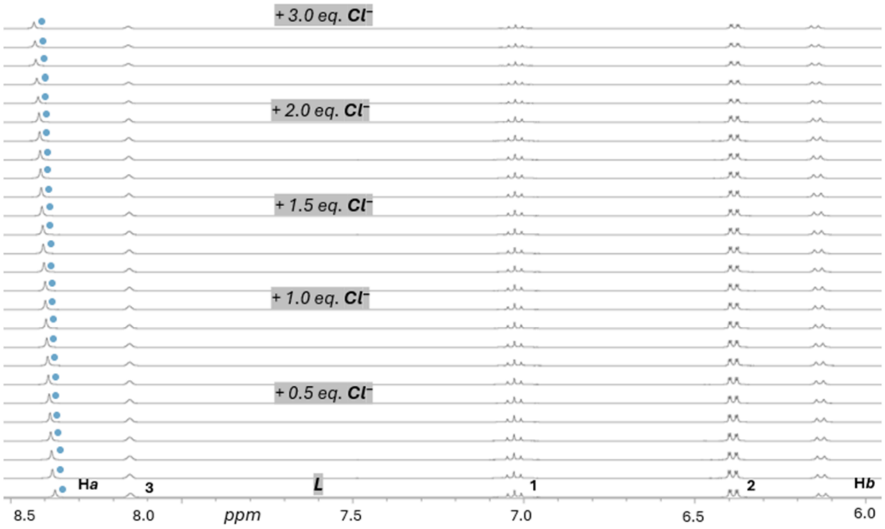

Figure S9. ^{1}H NMR titrations of **L** ($7.0 \cdot 10^{-3}$ mol·L^{-1}) recorded in DMSO-d_6 - 0.5% D_2O solution at 298 K by adding increasing amounts of Bu_4NCl (0.1 mol·L^{-1}) in DMSO-d_6. See Figure S7 for ^{1}H NMR labelling.

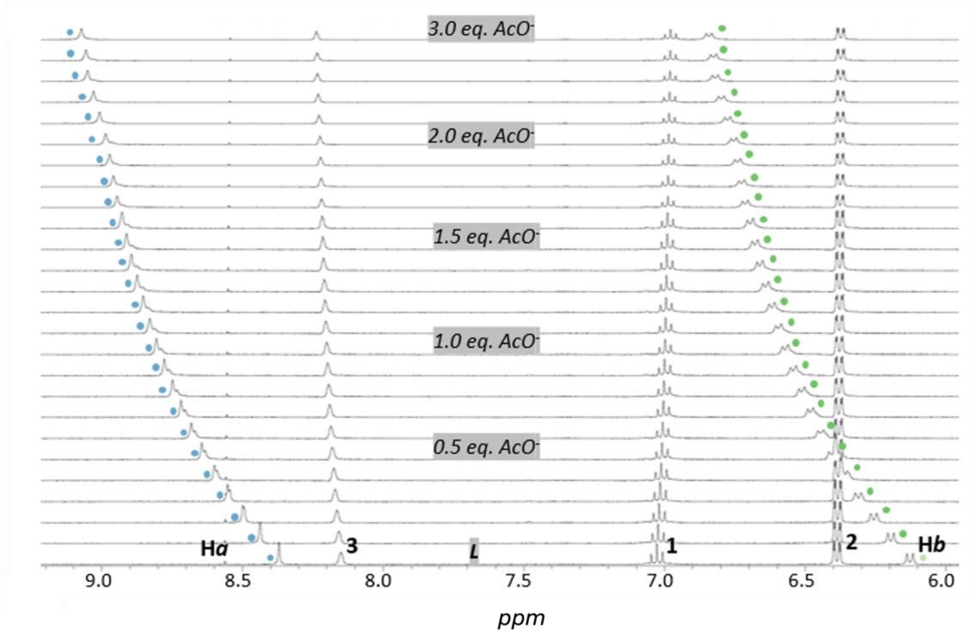

Figure S10. ^{1}H NMR titration of **L** ($7.0\cdot10^{-3}$ mol·L^{-1}) in DMSO-*d*6 - 0.5% D_2O solution at 298 K obtained by adding increasing amounts of Bu_4NAcO (0.1 mol·L^{-1}) in DMSO-*d*6. See Figure 1 for ^{1}H NMR labelling.

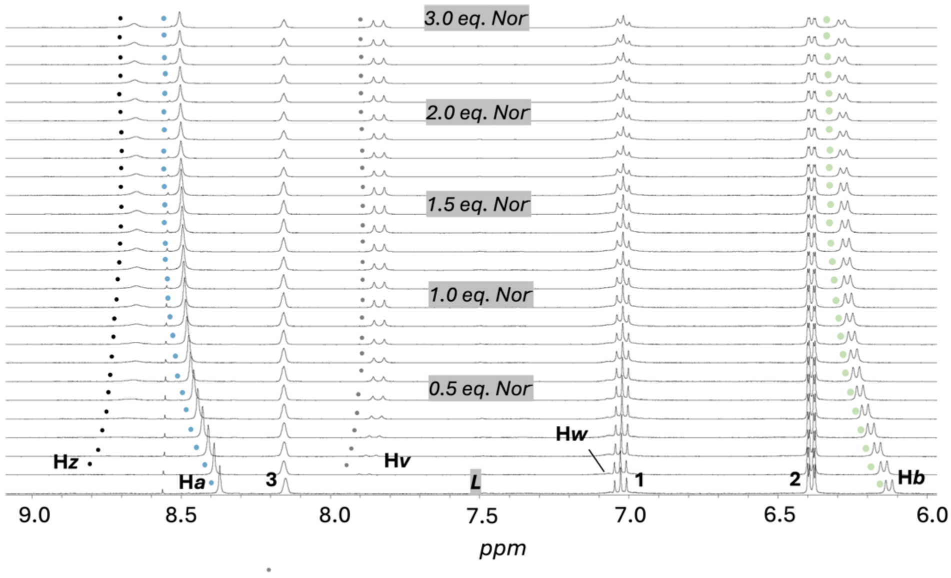

Figure S11. ^{1}H NMR titration of **L** ($7\cdot10^{-3}$ mol·L^{-1}) in DMSO-*d6* - 0.5% D_2O solution at 298 K obtained by adding increasing amounts of sodium norfloxacin (0.1 mol·L^{-1}) in DMSO-*d*6. See Figure 1 and Figure 6 for ^{1}H NMR labelling.

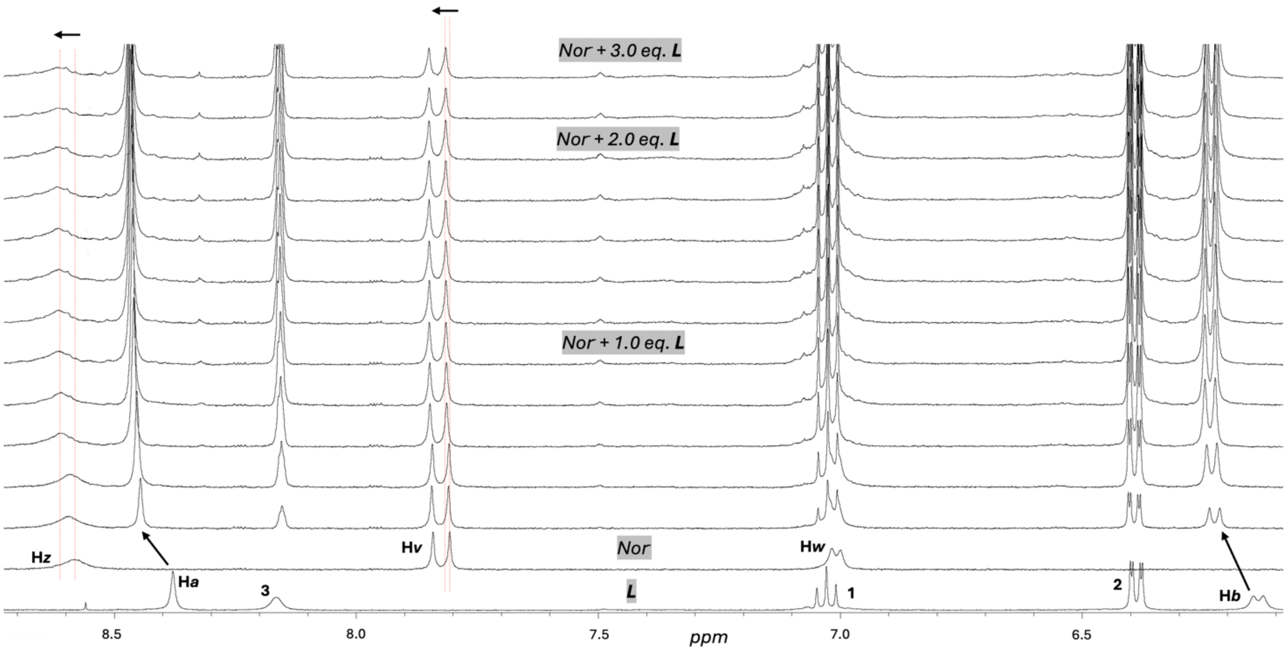

Figure S12. ^{1}H NMR titration of Nor^{-} ($6.5 \cdot 10^{-3}$ mol·L^{-1}) in DMSO-*d6* - 0.5% D_2O solution at 298 K obtained by adding increasing amounts of **L** ($6.0 \cdot 10^{-2}$ mol·L^{-1}) in DMSO-d_6. See Figure 1 and Figure 6 for ^{1}H NMR labelling.

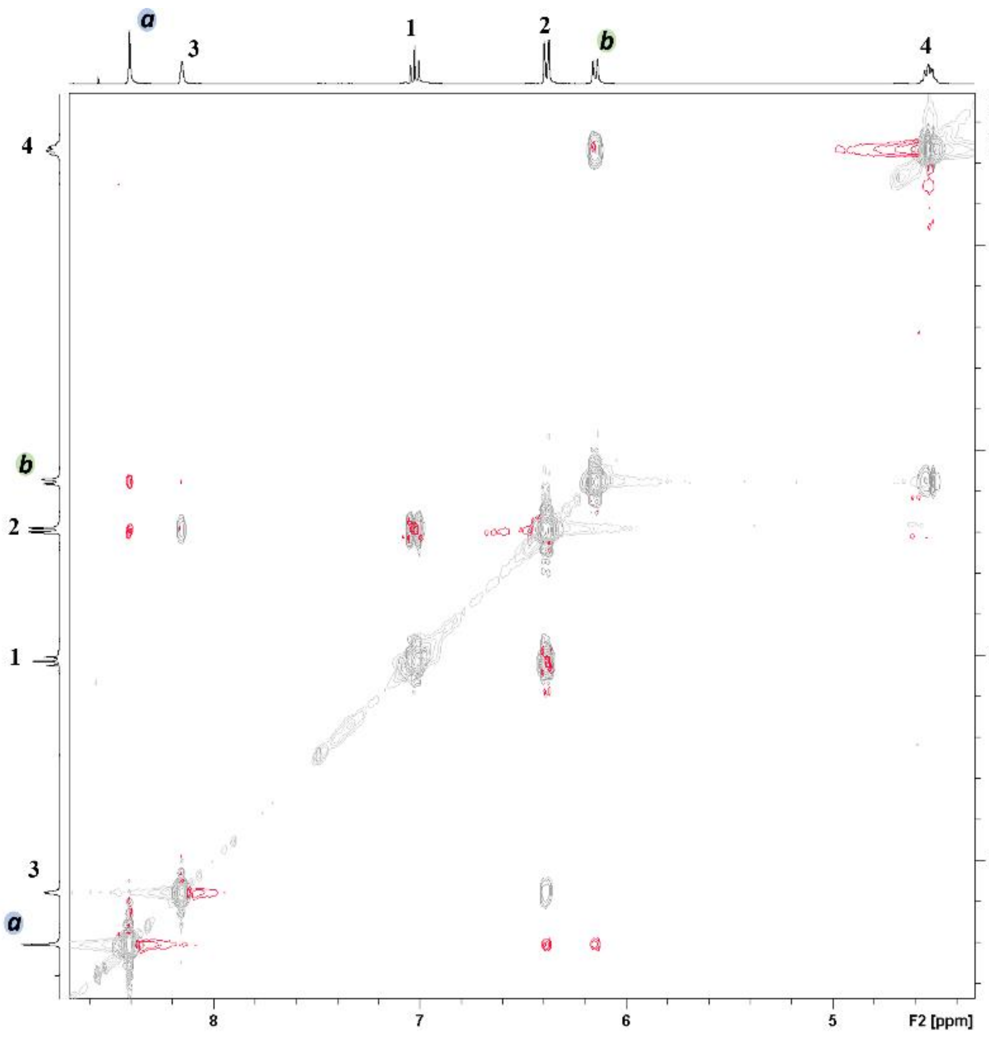

Figure S13. Superimposition of the ^{1}H COSY (grey spectrum) and ^{1}H NOESY (red spectrum) of **L** ($7.0 \cdot 10^{-3}$ mol·L^{-1}) in DMSO-d_6 - 0.5% D_2O solution at 298 K. See Figure S7 for ^{1}H NMR labelling.

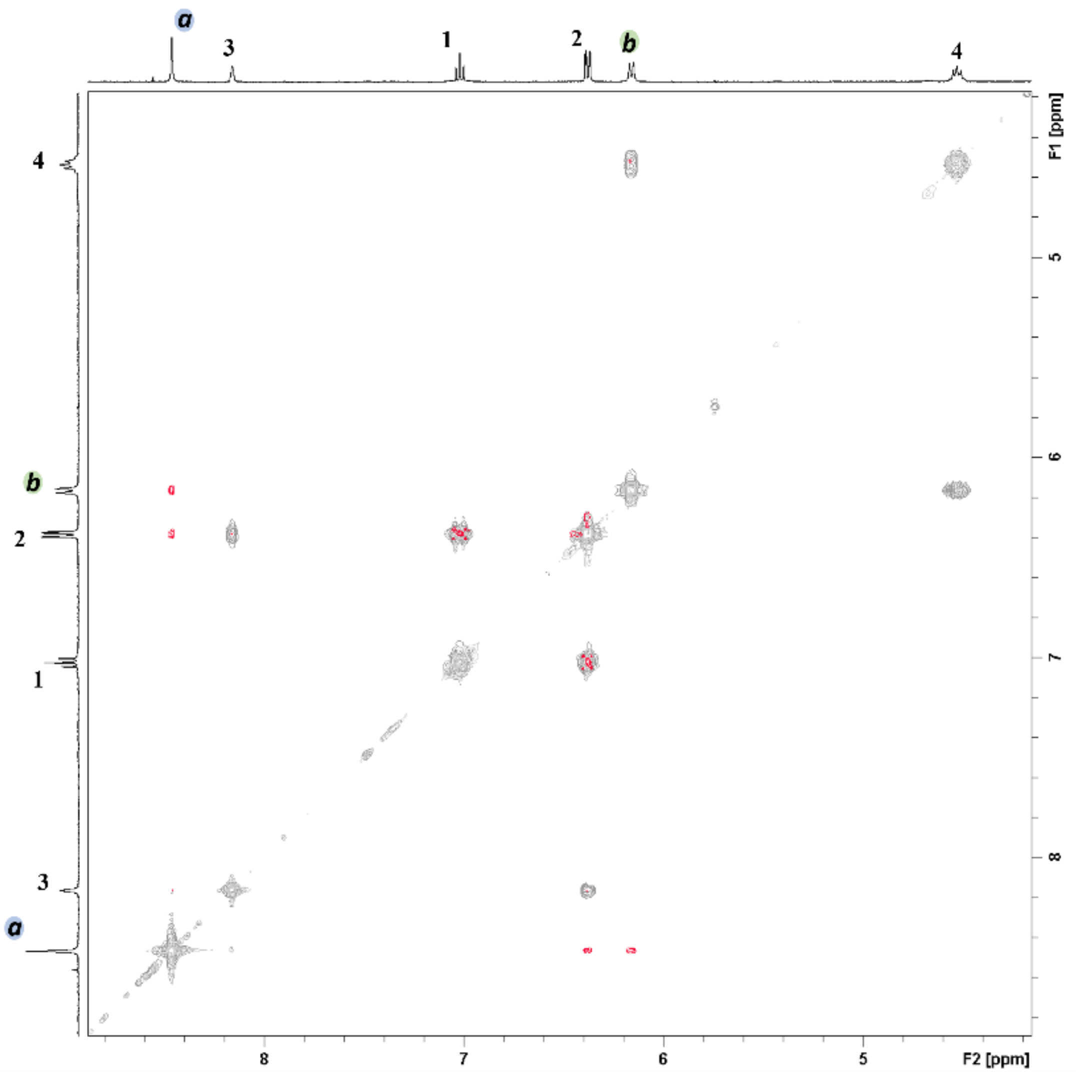

Figure S14. Superimposition of the ^{1}H COSY (grey spectrum) and ^{1}H NOESY (red spectrum) of **L** ($7.0 \cdot 10^{-3}$ mol·L^{-1}) in DMSO-d_6 - 0.5% D_2O solution at 298 K recorded after the addition of 1.0 equiv of TBACl in DMSO-d_6. See Figure S7 for ^{1}H NMR labelling.

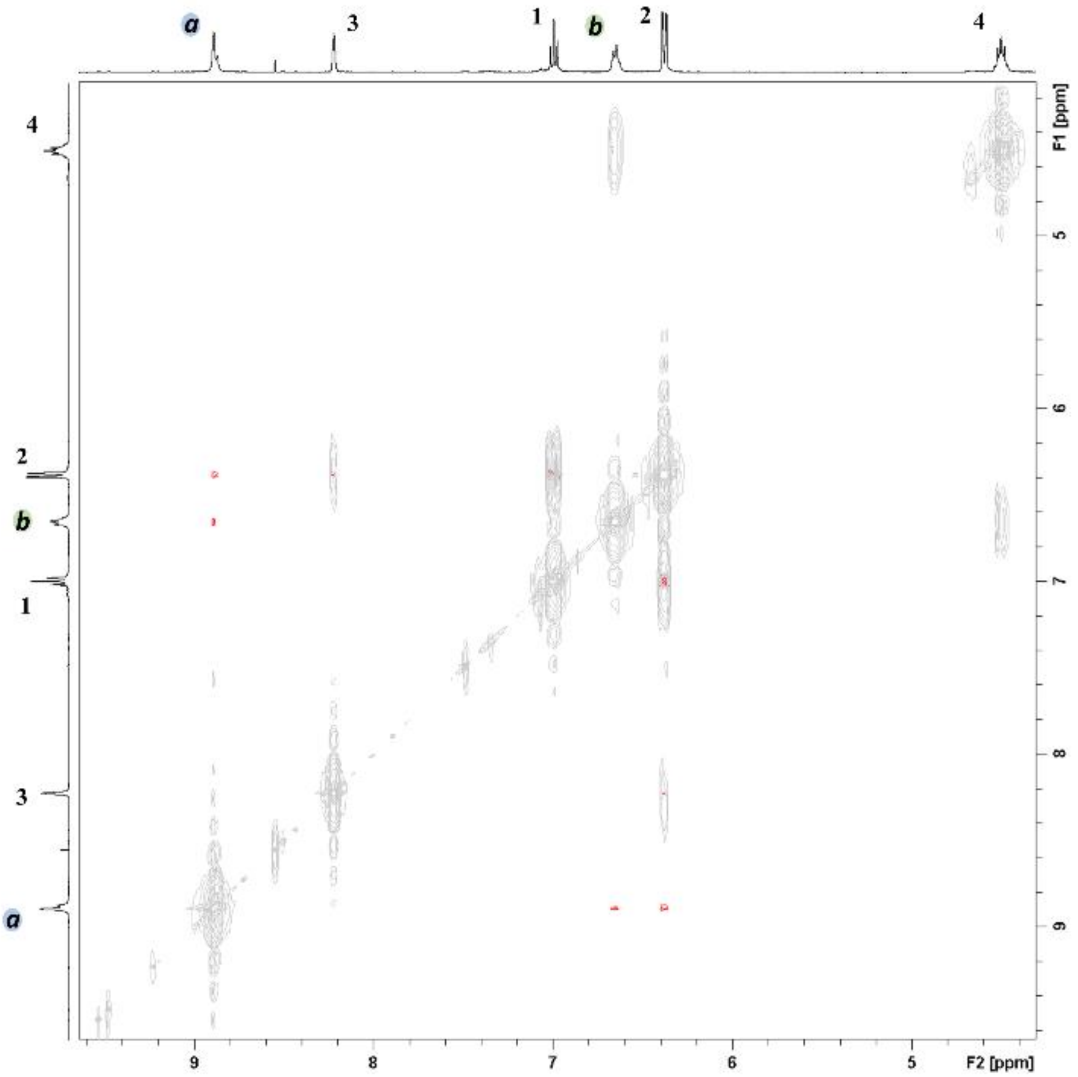

Figure S15. Superimposition of the ^{1}H COSY (grey spectrum) and ^{1}H NOESY (red spectrum) of **L** $7 \cdot 10^{-3}$ in DMSO-d_6 - 0.5% D_2O solution at 298 K recorded after the addition of 1.0 equiv of TBAAcO in DMSO-d_6. See Figure S7 for ^{1}H NMR labelling.

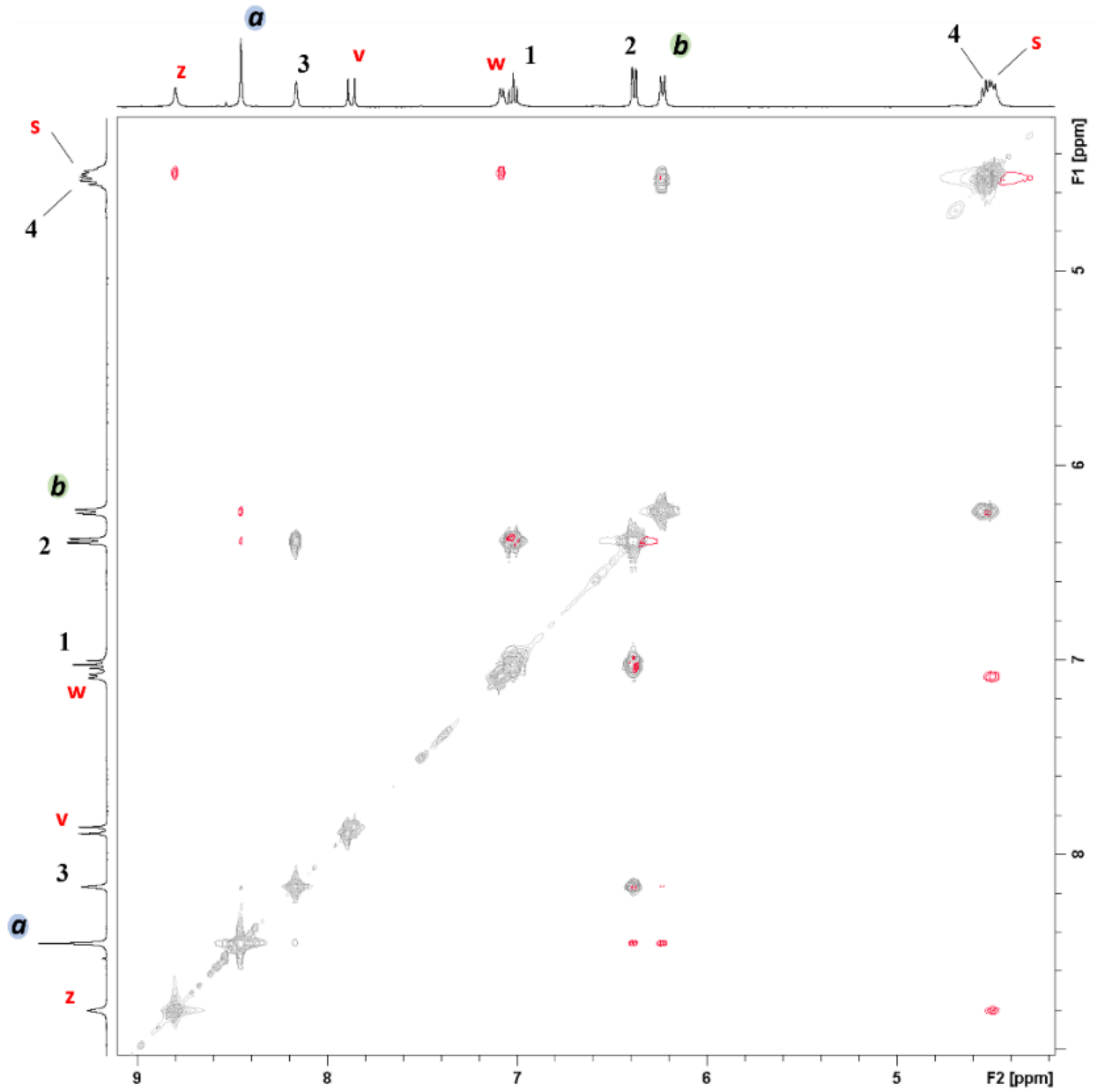

Figure S16. Superimposition of the ^{1}H Cosy (grey spectrum) and ^{1}H NOESY (red spectrum) of **L** ($7.0\cdot10^{-3}$ mol·L^{-1}) - 0.5% D_2O solution at 298 K recorded after the addition of 1.0 equiv of sodium norfloxacin in DMSO-*d*$_6$. See Figures S7 and 6 for ^{1}H NMR labelling of **L** and Nor^{-} respectively.

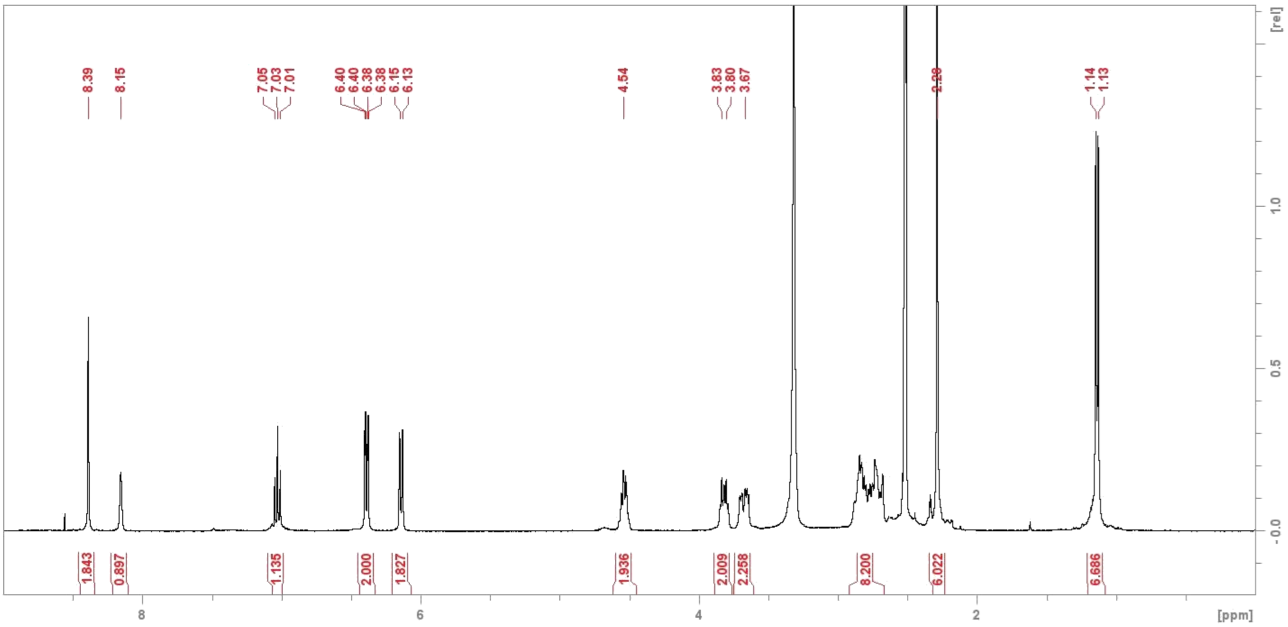

Figure S17. ^{1}H NMR spectrum of **L** in in DMSO-*d*$_6$.

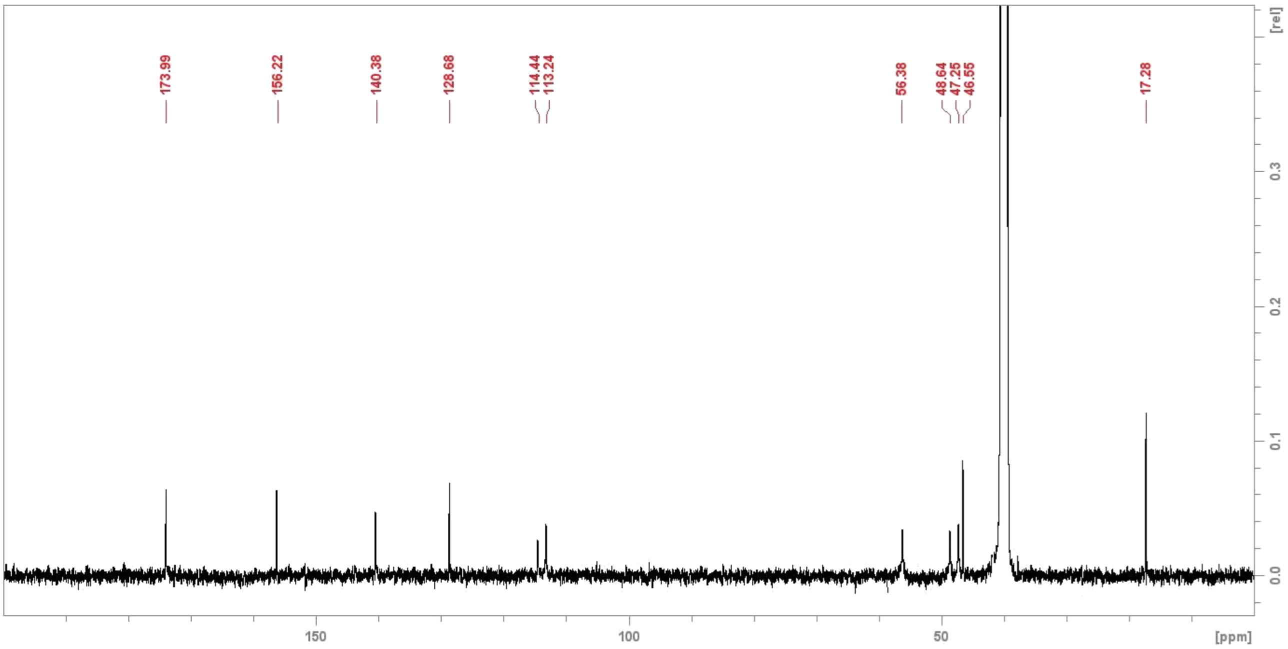

Figure S18. ^{13}C NMR spectrum of **L** in in DMSO-*d*$_6$.
